# Mobile devices for developing nursing students’ professional skills: scoping review ^1^

**DOI:** 10.1590/1518-8345.7383.4371

**Published:** 2024-09-23

**Authors:** Angélica Oliveira Veríssimo da Silva, Cristina Maria Correia Barroso Pinto, Rui Marques Vieira

**Affiliations:** 1 Universidade de Aveiro, Centro de Investigação em Didática e Tecnologia na Formação de Formadores, Departamento de Educação e Psicologia, Aveiro, Portugal.; 2 Escola Superior de Enfermagem do Porto, Centro de Investigação em Tecnologias e Serviços de Saúde e Rede de Investigação em Saúde, Porto, Portugal.

**Keywords:** Mobile Applications, Smartphone, Nursing students, Professional Competence, Professional Practice, Nurse’s Role

## Abstract

**Objective:**

to map the scientific literature on the use of mobile devices to develop the professional skills of nursing students.

**Method:**

this was a scoping review guided by JBI recommendations. Six databases and gray literature were included. The selection of studies was carried out through individual and peer assessment. Data were extracted based on an elaborate script and presented in a descriptive, tabular and graphical format.

**Results:**

264 studies were identified, of which 13 comprised the corpus of analysis. The studies were carried out mainly on the Asian continent. Interventions ranged from one to 12 weeks, with a predominance of the use of Apps. The skills addressed were mainly clinical skills, techniques and procedures inherent to nursing practice, followed by decision-making and problem-solving.

**Conclusion:**

the studies analyzed not only revealed the potential of mobile devices in the training context, but also highlighted their contribution to improving clinical capabilities, as they offer support for a more dynamic and effective approach to the learning process. The gap in knowledge appears in the still unexplored possibility of integrating different professional skills through a single digital educational tool.

## Highlights

**(1)** Positive repercussions of mobile devices on skills development.

**(2)** Knowledge gap is the absence of a tool that integrates several skills.

**(3)** Potential of mobile devices for improving clinical capabilities.

**(4)** Importance of integrating hard and soft skills into a single digital tool.

## Introduction

 In recent times there has been a growing concern with issues linked to safety and quality of care, as well as the need to minimize the occurrence of avoidable errors. From this perspective, different clinical situations and different contexts requires the nurse to demonstrates skills for good clinical judgment, problem solving and clinical decision-making ^(^ ^1^ ^-^ ^3^ ^)^ . For this reason, the Order of Nurses (ON) in Portugal, in its Nurse Competence Profile Regulation, establishes that in order to demonstrate professional competencies, a set of knowledge, skills and capabilities are necessary in the context of care ^(^ ^4^ ^)^ . 

 Therefore, competence is defined as “Competency is a holistic concept that includes knowledge, skills, attitudes and values” ^(^ ^5^ ^)^ . Being competent involves more than obtaining knowledge and skills, above all it involves mobilizing knowledge, skills, attitudes and values to respond to complex social and professional demands ^(^ ^5^ ^)^ . 

 In the context of healthcare, competence has a direct influence on the health and safety of patients ^(^ ^6^ ^)^ . To provide excellent care, much more is required than knowledge; the ability to think critically and apply knowledge in practice is imperative ^(^ ^7^ ^)^ . Therefore, the training context should be a place for transforming theoretical knowledge into practical knowledge. However, nursing training has faced several challenges, among which it is possible to mention the training environments that offer precarious opportunities to transfer the knowledge acquired in the classroom to clinical practice, sometimes due to the absence of clinical situations, sometimes due to failures in clinical supervision ^(^ ^8^ ^-^ ^9^ ^)^ . Added to this, there is a growing demand for a multiplicity of care ^(^ ^7^ ^,^ ^10^ ^)^ . Given this complex training context for nurses, establishing different contexts and strategies to bridge the gap between theory and practice are fundamental ^(^ ^8^ ^-^ ^9^ ^)^ . 

 Students in the 21 ^st^ century are in constant and increasing contact with digital technologies ^(^ ^11^ ^)^ . Nursing students also find themselves in this promising and challenging scenario ^(^ ^8^ ^,^ ^11^ ^)^ . Therefore, digital technologies are considered possible providers of important information for the decision-making process ^(^ ^8^ ^)^ . Thus, efforts are being made to incorporate digital technologies, hereinafter referred to as digital educational resources, in nursing training ^(^ ^8^ ^)^ . 

 For today’s globalized, computerized and constantly changing society, mobile learning stands out as the most promising digital learning technology ^(^ ^12^ ^-^ ^13^ ^)^ . Especially because books quickly become obsolete, while mobile devices provide up-to-date information ^(^ ^13^ ^)^ . Due to their ubiquitous nature, mobile devices ^(^ ^11^ ^)^ , especially smartphones, represent the most used digital technology worldwide ^(^ ^14^ ^)^ . In addition to their countless applicability, mobile devices are configured as facilitators of the teaching and learning process, as they enable flexibility, in other words, learning at any time and place ^(^ ^12^ ^,^ ^15^ ^-^ ^17^ ^)^ , which is why we are using the mobile learning concept. 

 Active, interactive and student-centered learning is considered to favor the development of nursing students’ professional skills ^(^ ^12^ ^,^ ^18^ ^)^ . Because it encourages students to think and act autonomously in their learning ^(^ ^12^ ^)^ . Such learning is easily made possible by using content from mobile devices ^(^ ^18^ ^)^ , especially mobile applications (Apps). Recent studies have shown that the use of mobile devices and their Apps in the context of nursing students’ learning contributed favorably to the development of professional skills ^(^ ^9^ ^,^ ^12^ ^,^ ^17^ ^-^ ^19^ ^)^ . Furthermore, they facilitated access to information, increased efficiency and optimized time ^(^ ^12^ ^)^ . A recent meta-analysis demonstrated that the use of mobile devices in the context of training nursing students promoted, in addition to knowledge, the improvement of professional skills, increased confidence, as well as satisfaction with learning ^(^ ^9^ ^)^ . It is also noteworthy that its use made it possible to reduce clinical errors ^(^ ^12^ ^)^ . In this way, the implementation of mobile devices and their Apps in nursing course curricula facilitates active learning and the promotion of care based on the most recent scientific evidence ^(^ ^13^ ^)^ . 

Furthermore, ensuring that nursing students receive quality training, possibilities for training contexts and strategies that facilitate the appropriation of knowledge and its applicability in clinical practice constitutes a major challenge for educators and managers. Faced with the challenge presented, fundamental questions emerge, which guided this research and they are: What is the impact of using mobile devices in the nursing training context? Which mobile devices are used to develop the professional skills of nursing students? Within this panorama, mobile learning emerges as a tool with great potential for developing nurses’ professional skills. Based on the urgent need to develop the professional skills of nursing students and the potential of mobile learning, the objective of this scoping review was to map the scientific literature on the use of mobile devices to develop the professional skills of nursing students.

## Method

### Study design

 The research protocol for this scoping review was registered in the Open Science Framework (OSF) ( https://doi.org/10.17605/OSF.IO/CESXK ). The synthesis of knowledge and evidence expressed by a scoping review follows the systematic recommendation of a literature review ^(^ ^20^ ^)^ . Its purpose is to map the main sources and types of evidence on a given topic, making it possible to identify basic concepts and theories, as well as potential gaps in knowledge ^(^ ^21^ ^)^ . 

 Methodological rigor and transparency must be present at all stages of a scoping review to enable the study to be replicated ^(^ ^22^ ^)^ . In order to comply with methodological rigor and transparency, this study follows the recommendations of the Joanna Briggs Institute (JBI) ^(^ ^23^ ^)^ , being conducted by the guidelines of the Checklist of the Preferred Reporting Items for Systematic Reviews and Meta-analyses extension for Scoping Reviews (PRISMA-ScR) ^(^ ^21^ ^)^ . 

In order to identify other similar studies or protocols, an initial search was carried out, in October 2023, on the OSF, as it is an open and free platform for depositing research and its respective protocols. No studies related to the proposed objective were identified, demonstrating the relevance of this review.

 Therefore, the steps proposed by JBI for designing a scoping review were followed: definition of the research question; research strategy; establishment of inclusion and exclusion criteria; study selection; data extraction; summary of results ^(^ ^23^ ^)^ . 

### Study setting

 The studies that make up this review were extracted from the following databases: Scopus, Web of Science (WoS), National Library of Medicine (PubMed), *Biblioteca do Conhecimento* Online (b-on) and SciELO. To integrate gray literature, searches for theses and dissertations were carried out in the Portuguese Open Access Scientific Repositories (POASR). 

### Period

Data searches took place between the months of October 2023 and January 2024. No time period was defined as a search criterion for eligible studies, since the objective is limited to mapping the maximum amount of published knowledge.

### Research strategy

The Population, Concept, Context (PCC) strategy was adopted, in which Population refers to nursing students, Concept refers to the development of professional skills and Context to mobile devices, to culminate in the elaboration of the research question: How are mobile devices used to develop the professional skills of nursing students?

### Eligibility criteria

After elaborating the research question, the eligibility criteria for inclusion and exclusion of studies were defined according to the PCC strategy. Thus, studies were included that: a) with regard to participants, include higher education nursing students; b) regarding the concept, studies that refer to the development of professional skills; c) regarding context, studies involving mobile devices, mobile learning or applications. Regarding the type of study, all primary, quantitative, qualitative and mixed studies were included, as well as secondary studies, namely literature reviews and gray literature. Furthermore, this review included studies published in Portuguese, English or Spanish, due to the fact that these are the languages the researchers are familiar with. Studies in open access format are added as an inclusion criterion. It is noteworthy that temporal limitation was not an exclusion criterion.

Studies in which the participants are nurses, students of technical courses or nursing assistants were excluded, as well as editorials, letters to the editor, study protocols, summaries and recommendations.

### Data collection

Having posed the research question and eligibility criteria, one of the researchers met with the university librarian to establish the search strategy. Therefore, a preliminary search was carried out in the Scopus database with the descriptors “nursing students”, “mobile devices” and “professional competence” to identify, in the titles and abstracts, the most used descriptors on the topic.

 Subsequently, the descriptors found were combined in different ways using Boolean operators AND and OR, resulting in Boolean phrases, as shown in Figure 1 , configuring the search strategy for the different databases. 

**Figure 1:** - Search strategy. Aveiro, Portugal, 2023

Data base	Search Strategy	Results
Scopus	( TITLE-ABS-KEY ( “nurs* education” OR “nurs* students” ) AND TITLE-ABS-KEY ( “mobile device*” OR “mobile technolog*” OR “mobile application*” OR “smartphone*” OR “mobile learning” ) AND TITLE-ABS-KEY ( “skill acquisition” OR “nurs* skills” OR “professional competence” OR “professionalism” OR “professional practice” OR “clinical competence” ) )	85
WoS	((TS=(“nurs* education” OR “nurs* students”)) AND TS=(“mobile device*” OR “mobile technolog*” OR “mobile application*” OR “smartphone*” OR “mobile learning” )) AND TS=(“skill acquisition” OR “nurs* skills” OR “professional competence” OR “professionalism” OR “professional practice” OR “clinical competence”)	27
PubMed	((“nursing education”[Title/Abstract] OR “nursing students”[Title/Abstract]) AND (“mobile devices”[Title/Abstract] OR “mobile technology”[Title/Abstract] OR “mobile application”[Title/Abstract] OR “smartphone”[Title/Abstract] OR “mobile learning”[Title/Abstract])) AND (“skill acquisition”[Title/Abstract] OR “nursing skills”[Title/Abstract] OR “professional competence”[Title/Abstract] OR “professionalism”[Title/Abstract] OR “professional practice”[Title/Abstract] OR “clinical competence”[Title/Abstract])	20
b-on	AB ( “nurs* education” OR “nurs* students” ) AND AB ( “mobile device*” OR “mobile technolog*” OR “mobile application*” OR “smartphone*” OR “mobile learning” ) AND AB ( “skill acquisition” OR “nurs* skills” OR “professional competence” OR “professionalism” OR “professional practice” OR “clinical competence” )	33
SciELO	#2 (ab:(estudante* de enfermagem OR educação em enfermagem)) AND (ab:(dispositivo* move* OR aprendiza* move* OR smartphone* OR app*))	14
POASR	Title “nursing students” AND “mobile devices” OR “mobile technology” OR “mobile application” OR “smartphone” OR “mobile learning” AND Abstract “nursing students” AND “mobile devices” OR “mobile technology” OR “mobile application” OR “smartphone” OR “mobile learning” AND Subject “nursing students” AND “mobile devices” OR “mobile technology” OR “mobile application” OR “smartphone” OR “mobile learning”	85

### Treatment and data analysis

 The results of the research carried out were exported to the Mendeley Reference Manager software version 2.80.1, and all duplicate studies were eliminated. Next, two independent reviewers read the titles and abstracts to verify compliance with the inclusion criteria and confirm the eligibility of the studies ^(^ ^24^ ^)^ . Any disagreements between the two reviewers were resolved by consensus or using a third reviewer. Studies that met the defined inclusion criteria were subjected to full text reading. Subsequently, the studies were imported into the Qualitative Data Analysis Software (webQDA) for their respective qualitative analysis. The data is presented in accordance with the objective of this review, in graphical, tabular and descriptive text format. 

After establishing the corpus, two independent reviewers performed data extraction using an instrument, according to JBI recommendations. The instrument was developed by the group of researchers to extract the following variables: author, study title, country in which the study was carried out, year of publication, type of study, objective, strategy/tool used, intervention time, competence to be developed and results achieved.

### Ethical aspects

The research only used public domain data, so it was not necessary to request for authorization from a Research Ethics Committee. It also emphasizes that copyright was respected through correct citation and bibliographic reference.

## Results

The search in the five databases and one repository made it possible to identify 264 potentially relevant studies. The application of exclusion criteria, namely language, type of study and lack of full text in Open Access format, allowed 112 studies to be excluded. Of the remaining 152 studies, 23 were excluded due to duplication. The remaining 129 were then subjected to reading of the titles and abstracts, thus, 93 were excluded. In the next phase, the remaining 36 studies were subjected to full reading, and of these, 23 were excluded. In view of this, through the established search strategy, the search in the databases and repository made it possible to identify 264 studies. The application of inclusion and exclusion criteria, plus the researchers’ thorough analysis, made it possible to identify a documentary sample of 13 studies, configuring the corpus of analysis for this review.

**Figure 2:**
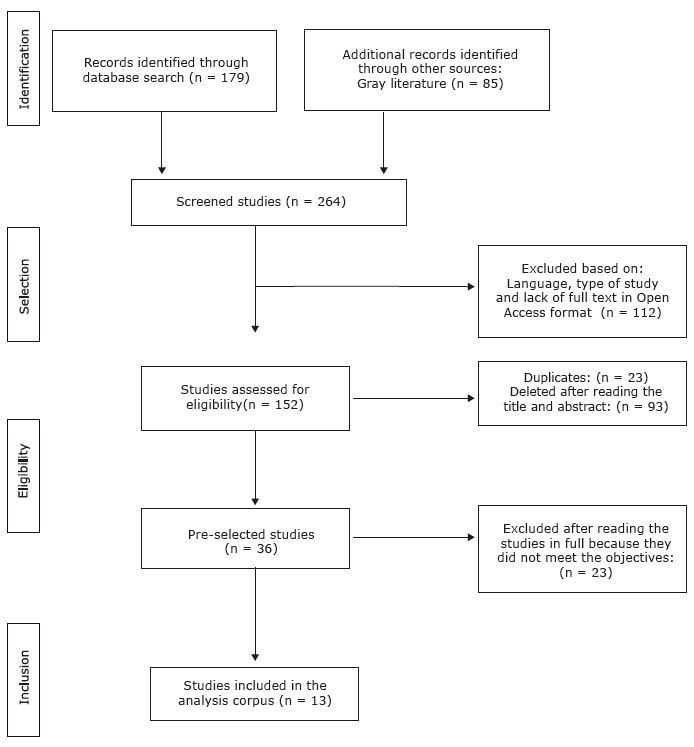
- PRISMA flowchart ^(^ ^24^ ^)^ . Aveiro, Portugal, 2023

 Eligible studies are represented in Figure 2 . 

The list of countries comprising this corpus is led by the Asian continent with nine studies, five from Korea, three from China and one from Iran. On the other hand, the European continent is represented by three studies, two from Norway and one from Finland. Finally, the American continent is represented in only one study, by Brazil.

 With regard to the distribution of studies by year of publication, it was found that four were published in 2018, representing the year with the highest number of publications (30.76%). Regarding the methodological aspects of the research included, the randomized clinical trial, present in four, and the quasi-experimental trial, represented by another four, stand out. The characterization of the studies regarding title, country, year of publication, type of study and objective is summarized in Figure 3 . 

 In order to systematize the results, the data were organized in the form of a table ( Figure 4 ) to highlight the strategy used in each study. Furthermore, the purpose of using it, that is, the professional competence that the strategy sought to develop, the intervention time and its respective results. 

**Figure 3:** - Characterization of studies regarding title, country, year of publication and type of study. Aveiro, Portugal, 2023

**ID**	**Title**	**Country/Year**	**Study design**	**Objective**
A1	A smartphone application to educate undergraduate nursing students about providing care for infant airway obstruction	Korea, 2017	Quasi-experimental	Develop a smartphone App and evaluate its effectiveness in terms of knowledge, skills and confidence of nursing students.
A2	Configuration of Mobile Learning Tools to Support Basic Physical Assessment in Nursing Education- Longitudinal Participatory Design Approach	Norway, 2021	Qualitative and longitudinal	Design a set of mobile learning tools to support nursing students’ learning.
A3	Effectiveness of mobile cooperation intervention on students’ clinical learning outcomes: A randomized controlled trial	Finland, 2018	Randomized and controlled clinical trial	To evaluate the effectiveness of mobile cooperation intervention to improve nursing students’ competence and self-efficacy.
A4	Effects of a skill demonstration video delivered by smartphone on facilitating nursing students’ skill competencies and self-confidence: A randomized controlled trial	China, 2018	Randomized and controlled clinical trial	Examining the effects of a smartphone video on nursing students’ competence and confidence.
A5	Effects of Nursing Students’ Practices using Smartphone Videos on Fundamental Nursing Skills, Self-efficacy, and Learning Satisfaction in South Korea	Korea, 2017	Quasi-experimental	Investigate the effects of learning through the use of video recording on smartphones in student self-assessment.
A6	Effects of using mobile device-based academic electronic medical records for clinical practicum by undergraduate nursing students- A quasi-experimental study.	Korea, 2018	Quasi-experimental	Examine the effect of an App on the clinical internship of nursing students.
A7	Evaluation of mobile learning for the clinical practicum in nursing education- application of the FRAME model	China, 2019	Experimental	To evaluate the perception of nursing students about the use of mobile devices in clinical practice.
A8	Factors associated with changes in students’ self-reported nursing competence after clinical rotations:A quantitative cohort study	Norway, 2023	Cohort Quantitative	Explore changes in nursing competency, factors associated with changes following clinical rotations, and whether a set of mobile learning tools supports changes in confident use of basic physical assessment skills.
A9	Use of digital applications in the medicament calculation education for nursing	Brazil, 2016	Experimental	Evaluate the influence of using digital applications in teaching medication calculations to nursing students.
A10	Mobile-Based Video Learning Outcomes in Clinical Nursing Skill Education: A Randomized Controlled Trial	Korea, 2016	Randomized and controlled clinical trial	To identify the effects of video clips on mobile devices on nursing students’ learning motivation, competence and satisfaction, and explore the relationships between these variables.
A11	Using Video Feedback Through Smartphone Instant Messaging in Fundamental Nursing Skills Teaching: Observational Study	China, 2019	Observational	Explore the possible effects of video feedback via smartphone messaging on teaching fundamental nursing skills to nursing students.
A12	The Effect of Team-based Training Through Smartphone Applications on Nursing Students’ Clinical Skills and Problem-Solving Ability	Iran, 2022	Quasi-experimental	To determine the effect of a team-based educational program using smartphone applications on the problem-solving ability and clinical capabilities of nursing students.
A13	The Effects of an Interactive Nursing Skills Mobile Application on Nursing Students’ Knowledge, Self-efficacy, and Skills Performance: A Randomized Controlled Trial	Korea, 2018	Randomized and controlled clinical trial	Evaluate the effect of a nursing skills App for nursing students.

**Figure 4:** - Characterization of the studies regarding the strategy used, the competence developed, the intervention time and the respective results. Aveiro, Portugal, 2023

**ID**	**Strategy**	**Competence**	**Intervention time**	**Results**
A1	App for clearing children’s airways (smartphone)	Clinical capacity (airway clearance)	6 weeks	Statistically significant difference from the experimental group in clinical capabilities and performance confidence. They reported greater satisfaction with learning.
A2	Mobile-Learning tools based on: Virtual clinical simulation, MOOC, videos and podcast	Clinical capacity (physical examination)	12 weeks	Students found the toolkit beneficial in supporting skills development.
A3	Study@Campus App: learning diary, cooperation between students and teachers and feedback (smartphone)	Clinical skills (Nurse Competence Scale) and self-efficacy	5 weeks	It revealed no statistical differences between the two groups. Significant effect on student satisfaction.
A4	Capabilities demonstration video (smartphone)	Knowledge, clinical skills and confidence (urinary catheterization)	2 weeks	Statistically significant differences in both knowledge, clinical skills and satisfaction with learning in the experimental group.
A5	Intramuscular medication administration practice video (smartphone)	Clinical capacity, self-efficacy and satisfaction (medication administration)	2 weeks	Significant and positive effects on improving competence and satisfaction with learning in the experimental group.
A6	Academic electronic medical record (AEMR) app (smartphone)	Decision making	4 weeks	The experimental group showed a significant increase in knowledge and skills. There was no statistically significant difference in critical thinking between the experimental and control groups. The experimental group showed greater satisfaction with learning.
A7	App 1- clinical skills assessment	Clinical capabilities	^*^	Participant satisfaction with mobile learning practice.
A8	App 2- clinical procedure videos	Clinical capacity and self-efficacy (physical examination)	8 weeks	Significant statistical differences in competence and confidence after using the toolkit.
A9	(tablet)	Calculation of medicines	^*^	It positively influenced learning and safety when calculating medications.
A10	Set of tools in the Canvas program (smartphone and tablet)	Clinical capacity (urinary catheterization)	3 weeks	Experimental group showed significantly higher levels of motivation and satisfaction with learning.
A11	CalcMed app (smartphone)	Clinical skills (bed making, aseptic procedures, vital signs, oxygen therapy)	8 weeks	The experimental group presented higher scores in the final exam of clinical skills and also greater satisfaction with the strategy used.
A12	Video urinary catheterization (smartphone)	Problem solving	12 weeks	Average scores increased more in the experimental group.
A13	Clinical skills demonstration videos for feedback (smartphone)	Knowledge, clinical skills and self-efficacy (vital signs, intravenous medication, gastric catheterization and endotracheal suctioning)	1 week	The experimental group presented a significantly higher value of knowledge, self-efficacy and clinical skills. They showed greater satisfaction with learning.

* It is not possible to identify the exact intervention time

## Discussion

The accessibility and versatility of mobile devices offer significant opportunities for learning. In view of this, this review aimed to map the scientific literature on the use of mobile devices to develop the professional skills of nursing students, identifying the different strategies used for this purpose.

 It is worth noting that the studies focused on the Asian continent, especially in Korea ^(^ ^18^ ^,^ ^25^ ^-^ ^28^ ^)^ and China ^(^ ^29^ ^-^ ^31^ ^)^ , which have emerged as global leaders in investments and technological advances. China, through a national program, has made great efforts towards scientific and technological development, with the aim of fostering a highly qualified workforce ^(^ ^32^ ^)^ . Furthermore, the analysis of the selected studies reveals a significant increase in interest in the topic, with a 69% increase in the number of articles published since 2018. 

 Furthermore, an important reflection refers to the intervention time, which varied between one ^(^ ^18^ ^)^ and 12 weeks ^(^ ^33^ ^-^ ^34^ ^)^ . Interestingly, the intervention time apparently did not determine the effectiveness of the strategy used, since the study carried out in just one week ^(^ ^18^ ^)^ demonstrated more satisfactory results in the experimental group compared to the control group. On the other hand, the study whose intervention was carried out over five weeks ^(^ ^35^ ^)^ did not show significant statistical differences between the experimental and control groups. This suggests that the relationship between intervention time and the acquisition of skills must be carefully considered, since skills can be developed through appropriate training contexts ^(^ ^3^ ^)^ . 

 In the meantime, students demonstrated high levels of satisfaction in ten studies when using strategies through their mobile devices ^(^ ^18^ ^,^ ^25^ ^-^ ^29^ ^,^ ^31^ ^,^ ^33^ ^,^ ^35^ ^)^ . A study in the United Kingdom revealed benefits such as acquiring knowledge and confidence, in addition to reducing anxiety in clinical practice ^(^ ^19^ ^)^ . A meta-analysis corroborated these findings, indicating that mobile learning improves students’ skills, knowledge, learning satisfaction and confidence ^(^ ^9^ ^)^ . 

 Regarding the type of intervention carried out and another relevant point, it is noted that of the 13 studies, seven used Apps ^(^ ^18^ ^,^ ^25^ ^,^ ^27^ ^,^ ^30^ ^,^ ^34^ ^-^ ^36^ ^)^ , four used videos to demonstrate clinical skills ^(^ ^26^ ^,^ ^28^ ^-^ ^29^ ^,^ ^31^ ^)^ and two used different tools on mobile devices, such as Massive Open Online Course (MOOC), videos, podcasts and tools in the Canvas program ^(^ ^33^ ^,^ ^37^ ^)^ . 

 Most Apps were specifically aimed at developing professional skills ^(^ ^18^ ^,^ ^25^ ^,^ ^27^ ^,^ ^30^ ^,^ ^36^ ^)^ . While a study in Finland used the Study@Campus App, which included a chat feature for interaction and immediate feedback ^(^ ^35^ ^)^ , in Iran, Telegram was used to provide educational content and share doubts ^(^ ^34^ ^)^ , proving to be effective in improving clinical capabilities and problem solving. This study strengthens the idea that communication through instant messaging, through social networks, is a space for sharing by promoting discussion in “real time” ^(^ ^38^ ^)^ . 

 Furthermore, videos stood out as an important strategy for developing professional skills, due to the possibility of continuous review of the content. Of the 13 studies, ten focused on the development of clinical skills, namely techniques and procedures that nurses must have knowledge, skills and abilities to carry out ^(^ ^18^ ^,^ ^25^ ^-^ ^26^ ^,^ ^28^ ^-^ ^31^ ^,^ ^33^ ^,^ ^35^ ^,^ ^37^ ^)^ , while three addressed skills such as decision-making ^(^ ^27^ ^)^ , problem-solving ^(^ ^34^ ^)^ and medication calculation ^(^ ^36^ ^)^ . Although the interventions did not deeply explore the potential of mobile devices for the development of multiple skills, focusing on developing clinical skills or other specific skills. 

 Considering the scope of the concept of competence, there was a need to segment between technical skills (hard skills) and transversal skills (soft skills) ^(^ ^39^ ^)^ . Soft skills are fundamental to promoting effective performance in different professional and social contexts ^(^ ^3^ ^)^ , in addition to encompassing cognitive, metacognitive, interpersonal, intellectual and practical capabilities, as well as ethical values ^(^ ^3^ ^,^ ^40^ ^)^ . While soft skills are considered “transferable”, as they can be applied in different situations and contexts, hard skills, related to specific technical and scientific knowledge, form the technical basis necessary to perform specialized functions ^(^ ^3^ ^,^ ^39^ ^)^ . A balanced combination of soft and hard skills is essential for professional excellence. 

 Therefore, effective clinical skills (hard skills) are essential for promoting safe and quality care ^(^ ^41^ ^-^ ^42^ ^)^ . Nursing students need to be encouraged to develop soft skills. To achieve this, they should be encouraged to think critically ^(^ ^1^ ^,^ ^43^ ^-^ ^44^ ^)^ and develop clinical judgment, problem-solving and decision-making skills ^(^ ^1^ ^,^ ^45^ ^-^ ^48^ ^)^ . These educational contexts encourage critical thinking and contribute to the training of more qualified and adaptable professionals ^(^ ^1^ ^,^ ^44^ ^)^ . Furthermore, the development of clinical judgment becomes essential so that nursing students can efficiently evaluate clinical scenarios for decision-making. In this sense, it is essential that training contexts are structured in a way that offers a holistic approach to the development of hard and soft skills, preparing students not only for technical demands, but also for the cognitive and decision-making challenges inherent to professional nursing practice ^(^ ^46^ ^,^ ^49^ ^)^ . Therefore, efforts should be oriented towards developing/strengthening critical thinking, clinical judgment, problem solving and decision making ^(^ ^1^ ^-^ ^3^ ^,^ ^44^ ^,^ ^50^ ^)^ . 

 In this context, mobile devices emerge as an economical, accessible and effective approach to improving professional skills ^(^ ^41^ ^)^ . They allow flexible learning environments, with access to content anytime and anywhere ^(^ ^25^ ^,^ ^41^ ^-^ ^42^ ^,^ ^51^ ^)^ . The possibility of continually revisiting knowledge, combined with familiarity and enjoyment of technology, makes mobile devices effective in developing professional skills ^(^ ^52^ ^-^ ^53^ ^)^ and student confidence ^(^ ^19^ ^,^ ^41^ ^)^ . The integration of teaching strategies through mobile devices can effectively engage and improve skills such as critical thinking, clinical judgment and decision-making ^(^ ^53^ ^-^ ^55^ ^)^ . In view of this, mobile devices not only streamline, but also complement the learning process. Furthermore, digital educational resources made available by mobile devices, such as MOOCs, Apps, videos, animations and games, can be used in both face-to-face and remote interfaces, and can contribute to more immersive and practical learning ^(^ ^56^ ^)^ . 

 Despite the potential of mobile devices, it is necessary to highlight possible negative aspects in the use of mobile devices in the training of nursing students ^(^ ^41^ ^,^ ^56^ ^-^ ^57^ ^)^ , such as distraction that can compromise efficiency and safety in care. A literature review carried out in Australia highlighted these negative impacts, including violations of patient safety and infection control ^(^ ^57^ ^)^ . 

In summary, the main contributions of this review are the positive outcomes evidenced in all the studies analyzed, confirming that mobile devices significantly influence the development of nursing students’ professional skills. However, it is necessary to conduct more comprehensive research to explore the synergy between professional and transversal skills, recognizing that nursing training encompasses not only technical capabilities, but also the holistic development of the student.

Finally, this study presents some limitations that must be considered, such as the small number of eligible studies and the relatively short intervention time observed in the studies analyzed. Therefore, longer interventions could provide a more detailed understanding of the effects of using mobile devices on the development of professional skills in nursing students.

## Conclusion

In conclusion, this literature review provided a mapping of published research that relates the use of mobile devices in the development of professional skills of nursing students. The studies analyzed not only revealed the inherent potential of these devices in the training environment, but have also significantly highlighted their contribution to improving students’ clinical capabilities. Furthermore, the convergence of results highlights the relevance and positive impact of mobile devices in the nursing training context, offering support for a more dynamic and effective approach to the learning process.

However, the review identified a gap in knowledge related to the untapped opportunity to integrate diverse professional skills through a single digital educational tool. This finding highlights the importance of future research and initiatives to explore and develop approaches that maximize the integration potential provided by mobile devices, promoting an effective synergy between different professional skills.

It is imperative that future studies focus not only on the development of isolated skills, but also on the balanced combination of technical and transversal skills (hard skills and soft skills). Furthermore, investigation into potential negative impacts such as distraction and patient safety must be further explored to ensure that the implementation of these technologies is safe and effective.

## References

[1] Silva A. O. V., Carvalho A. L. R. F., Vieira R. M., Pinto C. M. C. B. (2023). Estratégias de supervisão clínica, aprendizagem e pensamento crítico dos estudantes de Enfermagem. Rev Bras Enferm.

[2] Lau Y., Wang W. (2014). Development and Evaluation of a Learner-Centered Educational Summer Camp Program on Soft Skills for Baccalaureate Nursing Students. Nurse Educ.

[3] Widad A., Abdellah G. (2022). Strategies Used to Teach Soft Skills in Undergraduate Nursing Education: A Scoping Review. J Prof Nurs.

[4] Ordem dos Enfermeiros (PT) (2011). Regulamento do Perfil de Competências do Enfermeiro de Cuidados Gerais [Internet]. https://www.ordemenfermeiros.pt/media/8910/divulgar-regulamento-do-perfil_vf.pdf.

[5] Organisation for Economic Co-operation and Development (2019). Future of Education and Skills 2030. OECD Learning Compass 2030 [Internet]. https://www.oecd.org/education/2030-project/teaching-and-learning/learning/learning-compass-2030/OECD_Learning_Compass_2030_Concept_Note_Series.pdf.

[6] Hsu L. L., Hsieh S. I. (2013). Development and psychometric evaluation of the competency inventory for nursing students: A learning outcome perspective. Nurse Educ Today.

[7] Nemati-Vakilabad R., Mojebi M. R., Mostafazadeh P., Jafari M. J., Kamblash A. J., Shafaghat A. (2023). Factors associated with the critical thinking ability among nursing students: An exploratory study in Iran. Nurse Educ Pract.

[8] O’Connor S., Andrews T. (2015). Mobile Technology and Its Use in Clinical Nursing Education: A Literature Review. J Nurs Educ.

[9] Chen B., Wang Y., Xiao L., Xu C., Shen Y., Qin Q. (2021). Effects of mobile learning for nursing students in clinical education: A meta-analysis. Nurse Educ Today.

[10] Zuriguel-Pérez E., Falcó-Pegueroles A., Agustino-Rodríguez S., Gómez-Martín M. C., Roldán-Merino J., Lluch-Canut M. T. (2019). Clinical nurses’s critical thinking level according to sociodemographic and professional variables (Phase II): A correlational study. Nurse Educ Pract.

[11] Lee H., Min H., Oh S. M., Shim K. (2018). Mobile technology in undergraduate nursing education: A systematic review. Healthc Inform Res.

[12] Nikpeyma N., Zolfaghari M., Mohammadi A. (2021). Barriers and facilitators of using mobile devices as an educational tool by nursing students: a qualitative research. BMC Nurs.

[13] George T. P., Decristofaro C., Murphy P. F., Sims A. (2017). Student perceptions and acceptance of mobile technology in an undergraduate nursing program. Healthcare (Basel).

[14] Organização das Nações Unidas para a Educação, a Ciência e a Cultura. Diretrizes de políticas da UNESCO para a aprendizagem móvel [Internet] (2014). Organização das Nações Unidas para a Educação, a Ciência e a Cultura. Diretrizes de políticas da UNESCO para a aprendizagem móvel [Internet]. https://www.unesco.org/open-access/terms-use-ccbyncnd-port.

[15] Baars M., Khare S., Ridderstap L. (2022). Exploring Students’ Use of a Mobile Application to Support Their Self-Regulated Learning Processes. Front Psychol.

[16] Yalcinkaya T., Cinar Yucel S. (2023). Mobile learning in nursing education: A bibliometric analysis and visualization. Nurse Educ Pract.

[17] Kim J. H., Park H. (2019). Effects of Smartphone-Based Mobile Learning in Nursing Education: A Systematic Review and Meta-analysis. Asian Nurs Res.

[18] Kim H., Suh E. E. (2018). The Effects of an Interactive Nursing Skills Mobile Application on Nursing Students’ Knowledge, Self-efficacy, and Skills Performance: A Randomized Controlled Trial. Asian Nurs Res.

[19] O’Connor S., Andrews T. (2018). Smartphones and mobile applications (apps) in clinical nursing education: A student perspective. Nurse Educ Today.

[20] Armstrong R., Hall B. J., Doyle J., Waters E. (2011). Scoping the scope’ of a Cochrane review. J Public Health.

[21] Tricco A. C., Lillie E., Zarin W., O’Brien K. K., Colquhoun H., Levac D. (2018). PRISMA extension for scoping reviews (PRISMA-ScR): Checklist and explanation. Ann Intern Med.

[22] Arksey H., O’Malley L. (2005). Scoping studies: Towards a methodological framework. Int J Soc Res.

[23] Peters M. D. J., Godfrey C., McInerney P., Munn Z., Tricco A. C., Khalil H., Aromataris E., Munn Z. (2020). JBI Manual for Evidence Synthesis.

[24] Moher D., Liberati A., Tetzlaff J., Altman D. G., The PRISMA Group (2009). Preferred Reporting Items for Systematic Reviews and Meta-Analyses: The PRISMA Statement. PLoS Med.

[25] Kim S. J., Shin H., Lee J., Kang S. R., Bartlett R. (2017). A smartphone application to educate undergraduate nursing students about providing care for infant airway obstruction. Nurse Educ Today.

[26] Jeong H. S. (2017). Effects of nursing students’ practices using smartphone videos on fundamental nursing skills, self-efficacy, and learning satisfaction in South Korea. Eurasia J Math Sci Technol Educ.

[27] Choi M., Lee H. S., Park J. H. (2018). Effects of using mobile device-based academic electronic medical records for clinical practicum by undergraduate nursing students: A quasi-experimental study. Nurse Educ Today.

[28] Lee N. J., Chae S. M. M., Kim H., Lee J. H., Min H. J., Park D. E. (2016). Mobile-based video learning outcomes in clinical: Nursing skill education a randomized controlled trial. Comp Inform Nurs.

[29] Chuang Y. H., Lai F. C., Chang C. C., Wan H. T. (2018). Effects of a skill demonstration video delivered by smartphone on facilitating nursing students’ skill competencies and self-confidence: A randomized controlled trial study. Nurse Educ Today.

[30] Li K. C., Lee L. Y. K., Wong S. L., Yau I. S. Y., Wong B. T. M. (2019). Evaluation of mobile learning for the clinical practicum in nursing education: application of the FRAME model. J Comp Higher Educ.

[31] Yang X., Xie R. H., Chen S., Yu W., Liao Y., Krewski D. (2019). Using video feedback through smartphone instant messaging in fundamental nursing skills teaching: Observational study. JMIR MHealth UHealth.

[32] State Council (CHN) (2006). The National Medium-and Long-Term Program for Science and Technology Development (2006-2020) [Internet]. https://www.itu.int/en/ITUD/Cybersecurity/Documents/National_Strategies_Repository/China_2006.pdf.

[33] Egilsdottir H. Ö., Heyn L. G., Brembo E. A., Byermoen K. R., Moen A., Eide H. (2021). Configuration of mobile learning tools to support basic physical assessment in nursing education: Longitudinal participatory design approach. JMIR mHealth and uHealth.

[34] Motamed-Jahromi M., Eshghi F., Dadgar F., Nejadsadeghi E., Meshkani Z., Jalali T. (2022). The Effect of Team-based Training Through Smartphone Applications on Nursing Students’ Clinical Skills and Problem-Solving Ability. Shiraz E Med J.

[35] Strandell-Laine C., Saarikoski M., Löyttyniemi E., Meretoja R., Salminen L., Leino-Kilpi H. (2018). Effectiveness of mobile cooperation intervention on students’ clinical learning outcomes: A randomized controlled trial. J Adv Nurs.

[36] Pereira F. G. F., Caetano J. A., Frota N. M., Silva M. G. (2016). Use of digital applications in the medicament calculation education for nursing. Investig Educ Enferm.

[37] Egilsdottir H. Ö., Heyn L. G., Falk R. S., Brembo E. A., Byermoen K. R., Moen A. (2023). Factors associated with changes in students’ self-reported nursing competence after clinical rotations: a quantitative cohort study. BMC Med Educ.

[38] Willemse J. J., Bozalek V. (2015). Exploration of the affordances of mobile devices in integrating theory and clinical practice in an undergraduate nursing programme. Curationis.

[39] Laker D. R., Powell J. L. (2011). The differences between hard and soft skills and their relative impact on training transfer. Hum Resour Dev Q.

[40] Rao M. S. (2018). Soft skills: toward a sanctimonious discipline. On Horizon.

[41] Nikpeyma N., Zolfaghari M., Mohammadi A. (2021). Barriers and facilitators of using mobile devices as an educational tool by nursing students: a qualitative research. BMC Nurs.

[42] Chen B., Yang T., Wang Y., Xiao L., Xu C., Shen Y. (2021). Nursing students’ attitudes toward mobile learning: An integrative review. Int J Nurs Sci.

[43] Willers S., Jowsey T., Chen Y. (2021). How do nurses promote critical thinking in acute care? A scoping literature review. Nurse Educ Pract.

[44] Mártires A., Monteiro M. J., Rainho M. D. C., Castelo-Branco M. Z. (2019). Use of cooperative groups in the promotion of critical thinking skills in nursing students. Rev Lusofona Educ.

[45] Nunes J. G. P., Amendoeira J. J. P., Cruz D. A. L. M., Lasater K., Morais S. C. R. V., Carvalho E. C. (2020). Clinical judgment and diagnostic reasoning of nursing students in clinical simulation. Rev Bras Enferm.

[46] Hundial H. (2020). The Safe Care FrameworkTM: A practical tool for critical thinking. Nurse Educ Pract.

[47] Yang J., Zhou W. J., Zhou S. C., Luo D., Liu Q., Wang A. L. (2024). Integrated virtual simulation and face-to-face simulation for clinical judgment training among undergraduate nursing students: a mixed-methods study. BMC Med Educ.

[48] Pérez-Perdomo A., Zabalegui A. (2023). Teaching Strategies for Developing Clinical Reasoning Skills in Nursing Students: A Systematic Review of Randomised Controlled Trials. Healthcare.

[49] Oliveira L. B., Díaz L. J. R., Carbogim F. C., Rodrigues A. R. B., Püschel V. A. A. (2016). Effectiveness of teaching strategies on the development of critical thinking in undergraduate nursing students: A meta-analysis. Rev Esc Enferm.

[50] Alfaro-LeFevre R. (2014). Aplicação do processo de enfermagem. Fundamentos para o Raciocínio Clinico.

[51] Jang S., Suh E. E. (2022). Development and application of a mobile-based multimedia nursing competency evaluation system for nursing students: A mixed-method randomized controlled study. Nurse Educ Pract.

[52] Wirawan C. A., Arsa S. A. W. (2020). Development of guide Basic Life Support (BLS) application based on android to increase accuracy compression ritme and ventilation to handling of out hospital cardiac arrest. Babali Nurs Res.

[53] Baccin C. R. A., Dal Sasso G. T. M., Paixão C. A., Sousa P. A. F. (2020). Mobile Application as a Learning Aid for Nurses and Nursing Students to Identify and Care for Stroke Patients. Comp Inform Nurs.

[54] Carbogim F. C., Luiz F. S., Oliveira L. B., Braz P. R., Santos K. B., Püschel V. A. A. (2020). Effectiveness of a teaching model in a first aid course: a randomized clinical trial. Texto Contexto Enferm.

[55] Çatıker A., Büyüksoy G. D. B., Özdi̇l K. (2021). Is there a relationship between nursing students’ smartphone use, their fear of missing out and their care-related behaviour?. Nurse Educ Pract.

[56] Motta D. S., Cavalcante R. B., Dutra H. S., Coelho A. C. O., Pacheco Z. M. L., Santos K. B. (2022). Development and validation of technology for teaching basic life support in cardio-respiratory arrest. Cogitare Enferm.

[57] Ramjan L. M., Salamonson Y., Batt S., Kong A., McGrath B., Richards G. (2021). The negative impact of smartphone usage on nursing students: An integrative literature review. Nurse Educ Today.

