# Supplementary material for: Mobile devices for developing nursing students’ professional skills: scoping review
Source: Rev Lat Am Enfermagem. 2024 Sep 23;32:e4371. doi: 10.1590/1518-8345.7383.4371 (PMC11421521; doi:10.1590/1518-8345.7383.4371)
Supplement: Translation

# Translation: Dispositivos móviles para el desarrollo de las competencias profesionales de los estudiantes de enfermería: una revisión de alcance ^1^

**DOI:** 10.1590/1518-8345.7383.4370

**Authors:** Angélica Oliveira Veríssimo da Silva, Cristina Maria Correia Barroso Pinto, Rui Marques Vieira

**Affiliations:** 1 Universidade de Aveiro, Centro de Investigação em Didática e Tecnologia na Formação de Formadores, Departamento de Educação e Psicologia, Aveiro, Portugal.; 2 Escola Superior de Enfermagem do Porto, Centro de Investigação em Tecnologias e Serviços de Saúde e Rede de Investigação em Saúde, Porto, Portugal.

**Keywords:** Aplicaciones Móviles, Teléfono Inteligente, Estudiantes de Enfermería, Competencia Profesional, Práctica Profesional, Rol de la Enfermera

## Abstract

**Objetivo:**

mapear la literatura científica sobre la utilización de dispositivos móviles para el desarrollo de las competencias profesionales de los estudiantes de enfermería.

**Método:**

se trató de una revisión de alcance guiada por las recomendaciones del JBI. Se incluyeron seis bases de datos y la literatura gris. La selección de los estudios se realizó mediante evaluación individual y en pares. Los datos fueron extraídos con base en un guion elaborado, y presentados de forma descriptiva, tabular y gráfica.

**Resultados:**

se identificaron 264 estudios, de los cuales 13 compusieron el *corpus* de análisis. Los estudios se realizaron principalmente en el continente asiático. Las intervenciones variaron de 1 a 12 semanas, con predominio del uso de *Apps* . Las competencias abordadas fueron principalmente las capacidades clínicas, técnicas y procedimientos inherentes a la práctica de enfermería, seguidas por la toma de decisiones y resolución de problemas.

**Conclusión:**

los estudios analizados no solo revelaron las potencialidades de los dispositivos móviles en el contexto formativo, sino también destacaron su contribución para el perfeccionamiento de las capacidades clínicas, ya que ofrecen subsidios para un enfoque más dinámico y eficaz en el proceso de aprendizaje. La laguna en el conocimiento se perfila en la posibilidad aún no explorada de integración de diferentes competencias profesionales por medio de una única herramienta educativa digital.

## Puntos destacados

**(1)** Repercusiones positivas de los dispositivos móviles en el desarrollo de competencias.

**(2)** Laguna de conocimiento es la ausencia de una herramienta que integre varias competencias.

**(3)** Los dispositivos móviles tienen el potencial de mejorar las capacidades clínicas.

**(4)** Importancia de la integración de *hard* y *soft skills* en una única herramienta digital.

## Introducción

 En los últimos tiempos, ha habido una preocupación creciente por las cuestiones relacionadas con la seguridad y la calidad de los cuidados, así como con la necesidad de minimizar la ocurrencia de errores evitables. En esta perspectiva, se exige que en diversas situaciones clínicas y diferentes contextos, el enfermero demuestre competencias para el buen juicio clínico, la resolución de problemas y la toma de decisiones clínicas ^(^ ^1^ ^-^ ^3^ ^)^ . Por esta razón, la Orden de Enfermeros (OE) de Portugal, en su Reglamento del Perfil de Competencias del Enfermero, establece que para la demostración de las competencias profesionales es necesaria la movilización de un conjunto de conocimientos, habilidades y capacidades en el contexto de los cuidados ^(^ ^4^ ^)^ . 

 Así que, competencia se define como “ *A competency is a holistic concept that includes knowledge, skills, attitudes and values* ” ^(^ ^5^ ^)^ . Ser competente implica más que adquirir conocimientos y habilidades, sobre todo implica movilizar los conocimientos, las habilidades, las actitudes y los valores para responder a las complejas demandas sociales y profesionales ^(^ ^5^ ^)^ . 

 En el contexto de los cuidados de salud, la competencia tiene una influencia directa en la salud y seguridad de los pacientes ^(^ ^6^ ^)^ . Para la prestación de cuidados de excelencia, se requiere mucho más que el conocimiento; es imperativo la capacidad de pensar críticamente y aplicar el conocimiento en la práctica ^(^ ^7^ ^)^ . De esta manera, el contexto de formación debe ser un lugar de transformación del conocimiento teórico en práctico. No obstante, la formación en enfermería ha enfrentado diversos desafíos, entre los cuales se pueden citar los entornos formativos que ofrecen oportunidades escasas para transponer el conocimiento adquirido en el aula a la práctica clínica, ya sea por la ausencia de situaciones clínicas, ya sea por fallas en la supervisión clínica ^(^ ^8^ ^-^ ^9^ ^)^ . Además, se suma la creciente y exigente multiplicidad del cuidado ^(^ ^7^ ^,^ ^10^ ^)^ . Teniendo en cuenta este complejo contexto formativo del enfermero, es de fundamental importancia el establecimiento de diferentes contextos y de estrategias para colmar la brecha entre la teoría y la práctica ^(^ ^8^ ^-^ ^9^ ^)^ . 

 Los estudiantes del siglo XXI están en constante y creciente contacto con las tecnologías digitales ^(^ ^11^ ^)^ . En este escenario prometedor y desafiante también se encuentran los estudiantes de enfermería ^(^ ^8^ ^,^ ^11^ ^)^ . Luego, las tecnologías digitales se mencionan como facilitadoras del suministro de información importante para el proceso de toma de decisiones ^(^ ^8^ ^)^ . Así, se suman esfuerzos para la incorporación de las tecnologías digitales, en adelante denominadas recursos educativos digitales, en la formación en enfermería ^(^ ^8^ ^)^ . 

 Para la actual sociedad, globalizada, informatizada y en constante cambio, el aprendizaje móvil se destaca como la tecnología digital más prometedora para el aprendizaje ^(^ ^12^ ^-^ ^13^ ^)^ . Los libros, por otra parte, se vuelven obsoletos rápidamente, mientras que los dispositivos móviles proporcionan información actualizada ^(^ ^13^ ^)^ . Debido a su carácter omnipresente, los dispositivos móviles ^(^ ^11^ ^)^ , especialmente los *smartphones* , representan la tecnología digital más utilizada en el mundo ^(^ ^14^ ^)^ . Además de sus numerosas aplicaciones, los dispositivos móviles se configuran como facilitadores del proceso de enseñanza y aprendizaje, ya que permiten la flexibilidad, en otras palabras, el aprendizaje en cualquier momento y lugar ^(^ ^12^ ^,^ ^15^ ^-^ ^17^ ^)^ , por eso el concepto de aprendizaje móvil. 

 El aprendizaje activo, interactivo y centrado en el estudiante se señala como el que favorece el desarrollo de las competencias profesionales de los estudiantes de enfermería ^(^ ^12^ ^,^ ^18^ ^)^ . Esto se debe a que impulsa al estudiante a pensar y a actuar de forma autónoma en su aprendizaje ^(^ ^12^ ^)^ . Este aprendizaje se facilita fácilmente mediante el uso de contenidos provenientes de los dispositivos móviles ^(^ ^18^ ^)^ , especialmente de las aplicaciones móviles ( *Apps* ). Estudios recientes han demostrado que el uso de dispositivos móviles y de sus *Apps* en el contexto del aprendizaje de los estudiantes de enfermería contribuyó favorablemente al desarrollo de las competencias profesionales ^(^ ^9^ ^,^ ^12^ ^,^ ^17^ ^-^ ^19^ ^)^ . Además, facilitaron el acceso a la información, aumentaron la eficiencia y optimizaron el tiempo ^(^ ^12^ ^)^ . Un meta-análisis reciente demostró que el uso de dispositivos móviles en el contexto de la formación de estudiantes de enfermería promovió, además del conocimiento, el perfeccionamiento de las competencias profesionales, el aumento de la confianza, bien como de la satisfacción con el aprendizaje ^(^ ^9^ ^)^ . También se destaca que su uso permitió reducir errores clínicos ^(^ ^12^ ^)^ . De esta manera, la implementación de dispositivos móviles y sus *Apps* en los currículos de los cursos de enfermería facilita el aprendizaje activo y la promoción de cuidados basados en las evidencias científicas más recientes ^(^ ^13^ ^)^ . 

Además, garantizar que los estudiantes de enfermería reciban formación de calidad, posibilidades de contextos formativos y estrategias que faciliten la apropiación del conocimiento y su aplicabilidad en la práctica clínica constituye un gran desafío para los educadores y gestores. Ante el desafío presentado, emergen cuestiones fundamentales, las cuales orientaron esta investigación y son: ¿Cuál es el impacto de la utilización de dispositivos móviles en el contexto formativo de la enfermería? ¿Qué dispositivos móviles se utilizan para el desarrollo de las competencias profesionales de los estudiantes de enfermería? Dentro de este panorama, el aprendizaje móvil surge como una herramienta de gran potencial para el desarrollo de las competencias profesionales del enfermero. A partir de la urgente necesidad del desarrollo de las competencias profesionales de los estudiantes de enfermería y de las potencialidades del aprendizaje móvil, se estableció como objetivo para esta revisión de alcance mapear la literatura científica sobre la utilización de dispositivos móviles para el desarrollo de las competencias profesionales de los estudiantes de enfermería.

## Método

### Tipo del estudio

 El protocolo de investigación de esta *scoping review* fue registrado en el *Open Science Framework* (OSF) ( https://doi.org/10.17605/OSF.IO/CESXK ). La síntesis de conocimiento y evidencias expresadas por una revisión de alcance sigue la recomendación sistemática de una revisión de literatura ^(^ ^20^ ^)^ . Su finalidad es mapear las principales fuentes y tipos de evidencias sobre una determinada temática, permitiendo identificar conceptos y teorías fundamentales, así como potenciales lagunas en el conocimiento ^(^ ^21^ ^)^ . 

 El rigor metodológico y la transparencia deben estar presentes en todas las etapas de una revisión de alcance para permitir que el estudio sea replicado ^(^ ^22^ ^)^ . Con el fin de cumplir con el rigor metodológico y la transparencia, este estudio sigue las recomendaciones del *Joanna Briggs Institute* (JBI) ^(^ ^23^ ^)^ , siendo conducido por las directrices de la *Checklist del Preferred Reporting Items for Systematic Reviews and Meta-Analyses extension for Scoping Reviews* (PRISMA-ScR) ^(^ ^21^ ^)^ . 

Con el propósito de identificar otros estudios o protocolos similares, se realizó una búsqueda inicial, en octubre de 2023, en la OSF, por ser una plataforma abierta y gratuita para el depósito de investigaciones y sus respectivos protocolos. No se identificaron estudios relacionados con el objetivo propuesto, demostrando la pertinencia de esta revisión.

 Por lo tanto, se siguieron las etapas propuestas por el JBI para la concepción de una revisión de alcance: definición de la cuestión de investigación; estrategia de búsqueda; establecimiento de los criterios de inclusión y exclusión; selección de estudios; extracción de datos; síntesis de resultados ^(^ ^23^ ^)^ . 

### Escenario del estudio

 Los estudios que componen esta revisión fueron extraídos de las siguientes bases de datos: Scopus, *Web of Science* (WoS), *National Library of Medicine* (PubMed), *Biblioteca do Conhecimento* *Online* (b-on) y SciELO. Para la integración de la literatura gris se realizaron búsquedas de tesis y disertaciones en el Repositorio Científico de Acceso Abierto de Portugal (RCAAP). 

### Período

Las búsquedas de datos se llevaron a cabo entre los meses de octubre de 2023 y enero de 2024. No se delimitó un período temporal como criterio de búsqueda de estudios elegibles, ya que el objetivo es mapear la mayor cantidad de conocimiento publicado.

### Estrategia de búsqueda

Se adoptó la estrategia Población, Concepto, Contexto (PCC), en la cual Población se refiere a los estudiantes de enfermería, Concepto remite al desarrollo de competencias profesionales y Contexto a los dispositivos móviles, para culminar en la elaboración de la pregunta de investigación: ¿Cuáles son los dispositivos móviles utilizados para el desarrollo de las competencias profesionales de los estudiantes de enfermería?

### Criterios de elegibilidad

 Después de la elaboración de la pregunta de investigación, se definieron los criterios de elegibilidad para la inclusión y exclusión de los estudios según la estrategia PCC. Así, se incluyeron estudios que: a) en cuanto a los participantes, incluyan estudiantes de educación superior en enfermería; b) en cuanto al concepto, estudios que se refieren al desarrollo de competencias profesionales; c) en cuanto al contexto, estudios que involucren dispositivos móviles, aprendizaje móvil o aplicaciones. En lo que respecta al tipo de estudio, se contemplaron todos los estudios primarios, cuantitativos, cualitativos y mixtos, también estudios secundarios, como revisiones de literatura y literatura gris. Además, se incluyeron en esta revisión estudios publicados en portugués, inglés o español, debido a que son los idiomas con los que los investigadores están familiarizados. Se agregó como criterio de inclusión estudios en formato *open access* . Se destaca que la limitación temporal no fue un criterio de exclusión. 

Se excluyeron estudios en los que los participantes son enfermeros, estudiantes de cursos técnicos o auxiliares de enfermería, así como editoriales, cartas al editor, protocolos de estudio, resúmenes y recomendaciones.

### Recolección de datos

 Establecidas la pregunta de investigación y los criterios de elegibilidad, uno de los investigadores se reunió con la bibliotecaria de la universidad para establecer la estrategia de búsqueda. De esta manera, se realizó una búsqueda preliminar en la base de datos Scopus con los descriptores “ *nursing students* ”, “ *mobile devices* ” y “ *professional competence* ” para identificar en los títulos y resúmenes los descriptores más utilizados sobre el tema. 

 A continuación, los descriptores encontrados se combinaron de diferentes formas por medio de operadores booleanos *AND* y *OR* , resultando en las frases booleanas, como se muestra en la Figura 1 , configurándose como la estrategia de búsqueda para las diferentes bases de datos. 

**Figura 1:** - Estrategia de búsqueda. Aveiro, Portugal, 2023

Base de datos	Estrategia de búsqueda	Resultados
Scopus	( TITLE-ABS-KEY ( “nurs* education” OR “nurs* students” ) AND TITLE-ABS-KEY ( “mobile device*” OR “mobile technolog*” OR “mobile application*” OR “smartphone*” OR “mobile learning” ) AND TITLE-ABS-KEY ( “skill acquisition” OR “nurs* skills” OR “professional competence” OR “professionalism” OR “professional practice” OR “clinical competence” ) )	85
WoS	((TS=(“nurs* education” OR “nurs* students”)) AND TS=(“mobile device*” OR “mobile technolog*” OR “mobile application*” OR “smartphone*” OR “mobile learning” )) AND TS=(“skill acquisition” OR “nurs* skills” OR “professional competence” OR “professionalism” OR “professional practice” OR “clinical competence”)	27
PubMed	*((“nursing education”[Title/Abstract] OR “nursing students”[Title/Abstract]) AND (“mobile devices”[Title/Abstract] OR “mobile technology”[Title/Abstract] OR “mobile application” [Title/Abstract] OR “smartphone”[Title/Abstract] OR “mobile learning”[Title/Abstract])) AND (“skill acquisition”[Title/Abstract] OR “nursing skills”[Title/Abstract] OR “professional competence”[Title/Abstract] OR “professionalism”[Title/Abstract] OR “professional practice”[Title/Abstract] OR “clinical competence”[Title/Abstract])*	20
b-on	AB ( “nurs* education” OR “nurs* students” ) AND AB ( “mobile device*” OR “mobile technolog*” OR “mobile application*” OR “smartphone*” OR “mobile learning” ) AND AB ( “skill acquisition” OR “nurs* skills” OR “professional competence” OR “professionalism” OR “professional practice” OR “clinical competence” )	33
SciELO	#2 (ab:(estudante* de enfermagem *OR* educação em enfermagem )) *AND* (ab:(dispositivo* move* *OR* aprendiza* move* *OR* smartphone* *OR* *app* *))	14
RCAAP	*Title “nursing students” AND “mobile devices” OR “mobile technology” OR “mobile application” OR “smartphone” OR “mobile learning” AND Abstract “nursing students” AND “mobile devices” OR “mobile technology” OR “mobile application” OR “smartphone” OR “mobile learning” AND Subject “nursing students” AND “mobile devices” OR “mobile technology” OR “mobile application” OR “smartphone” OR “mobile learning”*	85

### Tratamiento y análisis de datos

 Los resultados de la investigación realizada se exportaron al *software Mendeley Reference Manager* versión 2.80.1, eliminando posteriormente todos los estudios duplicados. A continuación, dos revisores independientes realizaron la lectura de los títulos y de los resúmenes para verificar el cumplimiento de los criterios de inclusión y confirmar la elegibilidad de los estudios ^(^ ^24^ ^)^ . Las eventuales divergencias entre los dos revisores se resolvieron por consenso o recurriendo a un tercer revisor. Los estudios que respetaron los criterios de inclusión definidos se sometieron a la lectura del texto completo. Posteriormente, los estudios se importaron al *Qualitative Data Analysis Software* (webQDA) para su respectivo análisis cualitativo. Los datos se presentan de acuerdo con el objetivo de esta revisión, en formato gráfico, tabular y texto descriptivo. 

 Después del establecimiento del *corpus* , dos revisores independientes realizaron la extracción de datos mediante un instrumento según las recomendaciones de la JBI. El instrumento fue desarrollado por el grupo de investigadores para la extracción de las siguientes variables: autor, título del estudio, país de realización del estudio, año de publicación, tipo de estudio, objetivo, estrategia/herramienta utilizada, tiempo de intervención, competencia a ser desarrollada y resultados alcanzados. 

### Aspectos éticos

La investigación utilizó únicamente datos de dominio público, por lo que no fue necesario solicitar una opinión a un Comité de Ética en Investigación. Aún así, cabe destacar que los derechos de autor fueron respetados mediante la correcta citación y referencia bibliográfica.

## Resultados

 La búsqueda en las cinco bases de datos y un repositorio permitió identificar 264 estudios potencialmente relevantes. La aplicación de los criterios de exclusión, a saber, idioma, tipo de estudio y ausencia de texto completo en formato *Open Access* , permitió excluir 112 estudios. De los 152 estudios restantes, 23 se excluyeron debido a duplicidad. Los 129 estudios restantes se sometieron a la lectura de los títulos y resúmenes, excluyendo 93. En la fase siguiente, los 36 estudios restantes se sometieron a la lectura completa, excluyéndose 23. A la vista de esto, por medio de la estrategia de búsqueda establecida, la investigación en las bases de datos y repositorio permitió identificar 264 estudios. La aplicación de criterios de inclusión y de exclusión, sumado al análisis minucioso de los investigadores, permitió identificar una muestra documental de 13 estudios, configurando el *corpus* de análisis de esta revisión. 

 Los estudios elegibles se encuentran representados en la Figura 2 . 

**Figura 2:** - Diagrama PRISMA ^(^ ^24^ ^)^ . Aveiro, Portugal, 2023

 La lista de los países que constituyen este *corpus* está liderada por el continente asiático con 9 estudios, siendo 5 de Corea, 3 de China y 1 de Irán. En contraste, el continente europeo está representado por 3 estudios, siendo 2 de Noruega y 1 de Finlandia. Finalmente, el continente americano está representado solo por 1 estudio, de Brasil. 

 En lo que respecta a la distribución de los estudios por año de publicación, se verificó que en 2018 se publicaron 4, representando el año con mayor número de publicaciones (30,76%). En cuanto a los aspectos metodológicos de las investigaciones incluidas, se destaca el ensayo clínico aleatorizado, presente en 4, y el cuasi-experimental, representado por otros 4 estudios. La caracterización de los estudios en cuanto a título, país, año de publicación, tipo de estudio y objetivo se encuentra sintetizada en la Figura 3 . 

 Con el fin de sistematizar los resultados, los datos se organizaron en forma de cuadro ( Figura 4 ) para resaltar la estrategia utilizada en cada estudio. Además, se incluyó la finalidad de utilizarla, es decir, la competencia profesional que la estrategia buscó desarrollar, el tiempo de intervención y sus respectivos resultados. 

**Figura 3:** - Caracterización de los estudios en cuanto a título, país, año de publicación y tipo de estudio. Aveiro, Portugal, 2023

**ID**	**Título**	**País/Año**	**Tipo de Estudio**	**Objetivo**
A1	*A smartphone application to educate undergraduate nursing students about providing care for infant airway obstruction*	Corea, 2017	Cuasi-experimental	Desarrollar una *App* para *smartphone* y evaluar su eficacia en cuanto al conocimiento, a las capacidades y a la confianza de los estudiantes de enfermería.
A2	*Configuration of Mobile Learning Tools to Support Basic Physical Assessment in Nursing Education- Longitudinal Participatory Design Approach*	Noruega, 2021	Cualitativo y longitudinal	Diseñar un conjunto de herramientas de *mobile learning* para apoyar el aprendizaje de los estudiantes de enfermería.
A3	*Effectiveness of mobile cooperation intervention on students’ clinical learning outcomes: A randomized controlled trial*	Finlandia, 2018	Ensayo clínico aleatorizado y controlado	Evaluar la eficacia de la intervención de cooperación móvil para mejorar la competencia y la autoeficacia de los estudiantes de enfermería.
A4	*Effects of a skill demonstration video delivered by smartphone on facilitating nursing students’ skill competencies and self-confidence: A randomized controlled trial*	China, 2018	Ensayo clínico aleatorizado y controlado	Examinar los efectos de un video en *smartphone* en la competencia y en la confianza de los estudiantes de enfermería.
A5	*Effects of Nursing Students’ Practices using Smartphone Videos on Fundamental Nursing Skills, Self-efficacy, and Learning Satisfaction in South Korea*	Corea, 2017	Cuasi-experimental	Investigar los efectos del aprendizaje mediante el uso de grabación de video en *smartphones* en la autoevaluación de los estudiantes.
A6	*Effects of using mobile device-based academic electronic medical records for clinical practicum by undergraduate nursing students- A quasi-experimental study.*	Corea, 2018	Cuasi-experimental	Examinar el efecto de una *App* en la práctica clínica de los estudiantes de enfermería.
A7	*Evaluation of mobile learning for the clinical practicum in nursing education- application of the FRAME model*	China, 2019	Experimental	Evaluar la percepción de los estudiantes de enfermería sobre el uso de dispositivos móviles en la práctica clínica.
A8	*Factors associated with changes in students’ self-reported nursing competence after clinical rotations: A quantitative cohort study*	Noruega, 2023	Cuantitativo de cohorte	Explorar los cambios en la competencia de enfermería, los factores asociados a los cambios después de las rotaciones clínicas y si un conjunto de herramientas de aprendizaje móvil apoya los cambios en el uso confiado de las capacidades básicas de evaluación física.
A9	*Use of digital applications in the medicament calculation education for nursing*	Brasil, 2016	Experimental	Evaluar la influencia del uso de aplicaciones digitales en la enseñanza del cálculo de medicamentos para estudiantes de enfermería.
A10	*Mobile-Based Video Learning Outcomes in Clinical Nursing Skill Education: A Randomized Controlled Trial*	Corea, 2016	Ensayo clínico aleatorizado y controlado	Identificar los efectos de los videoclips en dispositivos móviles sobre la motivación para el aprendizaje, la competencia y la satisfacción de los estudiantes de enfermería, y explorar las relaciones entre estas variables.
A11	*Using Video Feedback Through Smartphone Instant Messaging in Fundamental Nursing Skills Teaching: Observational Study*	China, 2019	Observacional	Explorar los posibles efectos del *feedback* en video mediante mensajes en *smartphones* en la enseñanza de capacidades fundamentales de enfermería para estudiantes de enfermería.
A12	*The Effect of Team-based Training Through Smartphone Applications on Nursing Students’ Clinical Skills and Problem-Solving Ability*	Irán, 2022	Cuasi-experimental	Determinar el efecto de un programa educacional basado en equipo mediante aplicaciones de *smartphone* en la capacidad de resolución de problemas y en las capacidades clínicas de los estudiantes de enfermería.
A13	*The Effects of an Interactive Nursing Skills Mobile Application on Nursing Students’ Knowledge, Self-efficacy, and Skills Performance: A Randomized Controlled Trial*	Corea, 2018	Ensayo clínico aleatorizado y controlado	Evaluar el efecto de una *App* de capacidades de enfermería para estudiantes de enfermería.

**Figura 4:** - Caracterización de los estudios en cuanto a la estrategia utilizada, la competencia desarrollada, el tiempo de intervención y los respectivos resultados. Aveiro, Portugal, 2023

**ID**	**Estrategia**	**Competencia**	**Tiempo de intervención**	**Resultados**
A1	*App* para desobstrucción de vías aéreas de niños *(smartphone)*	Capacidad clínica (desobstrucción de vías aéreas)	6 semanas	Diferencia estadísticamente significativa del grupo experimental en las capacidades clínicas y en la confianza en el desempeño. Informaron mayor satisfacción con el aprendizaje.
A2	Herramientas *Mobile-Learning* basadas en: Simulación clínica virtual, *MOOC* , videos y *podcast*	Capacidad clínica (examen físico)	12 semanas	Los estudiantes consideraron el conjunto de herramientas beneficioso para apoyar el desarrollo de las competencias.
A3	*App Study@Campus :* diario de aprendizaje, cooperación entre estudiantes y profesores y *feedback (smartphone)*	Capacidades clínicas ( *Nurse Competence Scale* ) y autoeficacia	5 semanas	No reveló diferencias estadísticas entre los dos grupos. Efecto significativo en la satisfacción de los estudiantes.
A4	Video de demostración de capacidades *(smartphone)*	Conocimiento, capacidad clínica y confianza (cateterismo urinario)	2 semanas	Diferencias con significancia estadística tanto en el conocimiento, como en las capacidades clínicas y en la satisfacción con el aprendizaje del grupo experimental.
A5	Video de práctica de administración de medicación por vía intramuscular ( *smartphone* )	Capacidad clínica, autoeficacia y satisfacción (administración de medicamentos)	2 semanas	Efectos significativos y positivos en la mejora de la competencia y en la satisfacción con el aprendizaje del grupo experimewntal.
A6	*App Academic Electronic Medical Record* (AEMR) *(smartphone)*	Toma de decisiones	4 semanas	El grupo experimental presentó un aumento significativo en el dominio de conocimientos y competencias. No hubo diferencia con significancia estadística en el pensamiento crítico entre el grupo experimental y el grupo de control. El grupo experimental mostró mayor satisfacción con el aprendizaje.
A7	*App* 1: evaluación de capacidades clínicas *App* 2: videos de procedimientos clínicos	Capacidades clínicas	^*^	Satisfacción de los participantes con la práctica de aprendizaje móvil.
A8	Conjunto de herramientas en el programa Canvas *(smartphone e tablet)*	Capacidad clínica y autoeficacia (examen físico)	8 semanas	Diferencias estadísticamente significativas en la competencia y en la confianza después del uso del conjunto de herramientas.
A9	*App* CalcMed *(smartphone)*	Cálculo de medicamentos	^*^	Influyó positivamente en el aprendizaje y la seguridad en la realización del cálculo de medicamentos.
A10	Video de cateterismo urinario *(smartphone)*	Capacidad clínica (cateterismo urinario)	3 semanas	El grupo experimental presentó niveles significativamente más elevados de motivación y satisfacción con el aprendizaje.
A11	Videos de demostración de capacidades clínicas para *feedback (smartphone)*	Capacidades clínicas (arreglo de la cama, procedimientos asépticos, signos vitales, oxigenoterapia)	8 semanas	El grupo experimental presentó puntuaciones más altas en el examen final de las capacidades clínicas y, además, mayor satisfacción con la estrategia utilizada.
A12	*App Telegram* : materiales didácticos. *Team Based Learn* *(smartphone)*	Resolución de problemas	12 semanas	Las puntuaciones medias aumentaron más en el grupo experimental.
A13	*App* ICNS: simulación clínica *(smartphone)*	Conocimiento, capacidad clínica y autoeficacia (signos vitales, medicación intravenosa, sondaje gástrico y aspiración endotraqueal)	1 semana	El grupo experimental presentó un valor significativamente mayor de conocimiento, autoeficacia y capacidades clínicas. Mostraron mayor satisfacción con el aprendizaje.

* No es posible identificar el tiempo exacto de intervención

## Discusión

La accesibilidad y versatilidad de los dispositivos móviles ofrecen oportunidades significativas para el aprendizaje. Esta revisión tuvo como objetivo mapear la literatura científica sobre la utilización de los dispositivos móviles para el desarrollo de las competencias profesionales de los estudiantes de enfermería, identificando las diferentes estrategias utilizadas para ese fin.

 Cabe señalar que los estudios se concentraron en el continente asiático, especialmente en Corea ^(^ ^18^ ^,^ ^25^ ^-^ ^28^ ^)^ y en China ^(^ ^29^ ^-^ ^31^ ^)^ , que emergieron como líderes globales en inversiones y avances tecnológicos. China, por intermedio de un programa nacional, ha emprendido grandes esfuerzos para el desarrollo científico y tecnológico, con el objetivo de fomentar una fuerza laboral altamente calificada ^(^ ^32^ ^)^ . Además, el análisis de los estudios seleccionados revela un aumento significativo en el interés por el tema, con un crecimiento del 69% en el número de artículos publicados a partir de 2018. 

 Además, una reflexión importante se refiere al tiempo de intervención, que varió entre 1 ^(^ ^18^ ^)^ a 12 semanas ^(^ ^33^ ^-^ ^34^ ^)^ . Curiosamente, el tiempo de intervención aparentemente no determinó la eficacia de la estrategia utilizada, ya que el estudio realizado en solo 1 semana ^(^ ^18^ ^)^ demostró resultados más satisfactorios del grupo experimental en comparación con el grupo de control. En contraste, el estudio cuya intervención se realizó en 5 semanas ^(^ ^35^ ^)^ no mostró diferencias estadísticas significativas entre los grupos experimental y control. Esto sugiere que la relación entre el tiempo de intervención y la adquisición de competencias debe ser cuidadosamente considerada, ya que las competencias pueden desarrollarse mediante contextos formativos apropiados ^(^ ^3^ ^)^ . 

 En este ínterin, los estudiantes demostraron niveles elevados de satisfacción en diez estudios al utilizar estrategias por medio de sus dispositivos móviles ^(^ ^18^ ^,^ ^25^ ^-^ ^29^ ^,^ ^31^ ^,^ ^33^ ^,^ ^35^ ^)^ . Un estudio en el Reino Unido reveló beneficios como la adquisición de conocimiento y confianza, además de la reducción de la ansiedad en la práctica clínica ^(^ ^19^ ^)^ . Un meta-análisis corroboró estos hallazgos, indicando que el aprendizaje móvil mejora las competencias, los conocimientos, la satisfacción con el aprendizaje y la confianza de los estudiantes ^(^ ^9^ ^)^ . 

 Sobre el tipo de intervención realizada es otro punto relevante. Se nota que de los 13 estudios, siete utilizaron *Apps* ^(^ ^18^ ^,^ ^25^ ^,^ ^27^ ^,^ ^30^ ^,^ ^34^ ^-^ ^36^ ^)^ , cuatro utilizaron videos para demostración de capacidades clínicas ^(^ ^26^ ^,^ ^28^ ^-^ ^29^ ^,^ ^31^ ^)^ y dos utilizaron diferentes herramientas en dispositivos móviles, como *Massive Open Online Course* (MOOC), videos, podcasts y herramientas en el programa Canvas ^(^ ^33^ ^,^ ^37^ ^)^ . 

 La mayoría de las *Apps* se enfocaron específicamente en el desarrollo de competencias profesionales ^(^ ^18^ ^,^ ^25^ ^,^ ^27^ ^,^ ^30^ ^,^ ^36^ ^)^ . Mientras que un estudio en Finlandia utilizó la *App Study@Campus* , que incluía un recurso de *chat* para interacción y *feedback* inmediato ^(^ ^35^ ^)^ , en Irán, *Telegram* se utilizó para la distribución de contenidos didácticos y el intercambio de dudas ^(^ ^34^ ^)^ , mostrándose eficaz en el perfeccionamiento de capacidades clínicas y resolución de problemas. Este estudio fortalece la idea de que la comunicación por medio de mensajes instantáneos en las redes sociales se configura como un espacio de intercambio al promover la discusión en “tiempo real” ^(^ ^38^ ^)^ . 

 Además de que, los videos se destacaron como una estrategia importante para el desarrollo de competencias profesionales, debido a la posibilidad de revisión continua del contenido. De los 13 estudios, diez se centraron en el desarrollo de competencias clínicas, a saber, técnicas y procedimientos que los enfermeros deben conocer, habilidades y capacidades para realizarlos ^(^ ^18^ ^,^ ^25^ ^-^ ^26^ ^,^ ^28^ ^-^ ^31^ ^,^ ^33^ ^,^ ^35^ ^,^ ^37^ ^)^ , mientras que tres abordaron competencias como toma de decisiones ^(^ ^27^ ^)^ , resolución de problemas ^(^ ^34^ ^)^ y cálculo de medicamentos ^(^ ^36^ ^)^ . No obstante que las intervenciones no exploraron profundamente las potencialidades de los dispositivos móviles para el desarrollo de múltiples competencias, enfocándose en desarrollar capacidades clínicas u otras competencias específicas. 

 Considerando la amplitud del concepto de competencia, hubo la necesidad de segmentación entre competencias técnicas ( *hard skills* ) y transversales ( *soft skills* ) ^(^ ^39^ ^)^ . Las *soft skills* son fundamentales para promover el desempeño eficaz en diversos contextos profesionales y sociales ^(^ ^3^ ^)^ , además de incluir las capacidades cognitivas, metacognitivas, interpersonales, intelectuales y prácticas, así como los valores éticos ^(^ ^3^ ^,^ ^40^ ^)^ . Mientras que las *soft skills* se consideran “transferibles”, ya que pueden aplicarse en diversas situaciones y contextos, las *hard skills* , relacionadas con los conocimientos técnicos y científicos específicos, forman la base técnica necesaria para desempeñar funciones especializadas ^(^ ^3^ ^,^ ^39^ ^)^ . La combinación equilibrada de *soft skills* y *hard skills* es esencial para la excelencia profesional. 

 Así que, capacidades clínicas ( *hard skills* ) eficaces son esenciales para la promoción de cuidados seguros y de calidad ^(^ ^41^ ^-^ ^42^ ^)^ . Los estudiantes de enfermería necesitan ser estimulados al desarrollo de las *soft skills* . Para esto, deben ser incentivados a pensar de forma crítica ^(^ ^1^ ^,^ ^43^ ^-^ ^44^ ^)^ y desarrollar las competencias de juicio clínico, resolución de problemas y toma de decisiones ^(^ ^1^ ^,^ ^45^ ^-^ ^48^ ^)^ . Contextos educativos estos que estimulan el pensamiento crítico contribuyen a la formación de profesionales más capacitados y adaptables ^(^ ^1^ ^,^ ^44^ ^)^ . Además, el desarrollo del juicio clínico se torna fundamental para que los estudiantes de enfermería puedan evaluar eficientemente los escenarios clínicos para la toma de decisiones. En este sentido, es fundamental que los contextos formativos sean estructurados de manera que ofrezcan un enfoque holístico en el desarrollo de las *hard* y *soft skills* , preparando a los estudiantes no solo para las demandas técnicas, sino también para los desafíos cognitivos y decisorios inherentes a la práctica profesional en enfermería ^(^ ^46^ ^,^ ^49^ ^)^ . De esta forma, los esfuerzos deben orientarse hacia el desarrollo/fortalecimiento del pensamiento crítico, del juicio clínico, de la resolución de problemas y de la toma de decisiones ^(^ ^1^ ^-^ ^3^ ^,^ ^44^ ^,^ ^50^ ^)^ . 

 En este contexto, los dispositivos móviles surgen como un enfoque económico, accesible y eficaz para mejorar las competencias profesionales ^(^ ^41^ ^)^ . Permiten un entorno de aprendizaje flexible, con acceso a los contenidos en cualquier momento y lugar ^(^ ^25^ ^,^ ^41^ ^-^ ^42^ ^,^ ^51^ ^)^ . La posibilidad de revisar continuamente el conocimiento, combinada con la familiaridad y el gusto por la tecnología, hace que los dispositivos móviles sean eficaces en el desarrollo de competencias profesionales ^(^ ^52^ ^-^ ^53^ ^)^ y en la confianza de los estudiantes ^(^ ^19^ ^,^ ^41^ ^)^ . La integración de estrategias de enseñanza por medio de dispositivos móviles puede efectivamente involucrar y mejorar competencias como el pensamiento crítico, el juicio clínico y la toma de decisiones ^(^ ^53^ ^-^ ^55^ ^)^ . En vista de ello, los dispositivos móviles no solo dinamizan, sino que también complementan el proceso de aprendizaje. Además, los recursos educativos digitales disponibles por intermedio de dispositivos móviles, como MOOC, *Apps* , videos, animaciones y juegos, pueden ser utilizados tanto en entornos presenciales como remotos, contribuyendo a un aprendizaje más inmersivo y práctico ^(^ ^56^ ^)^ . 

 A pesar de las potencialidades de los dispositivos móviles, es necesario resaltar posibles aspectos negativos en su utilización en la formación de los estudiantes de enfermería ^(^ ^41^ ^,^ ^56^ ^-^ ^57^ ^)^ , como la distracción que puede comprometer la eficiencia y la seguridad en la atención. Una revisión de literatura realizada en Australia destacó estos impactos negativos, incluyendo violaciones de la seguridad del paciente y en el control de infecciones ^(^ ^57^ ^)^ . 

En síntesis, las principales contribuciones de esta revisión son los resultados positivos evidenciados en todos los estudios analizados, confirmando que los dispositivos móviles influyen significativamente en el desarrollo de las competencias profesionales de los estudiantes de enfermería. No obstante, es necesario realizar investigaciones más amplias para explorar la sinergia entre las competencias profesionales y transversales, reconociendo que la formación en enfermería abarca no solo las habilidades técnicas, sino también el desarrollo holístico del estudiante.

Por último, este estudio presenta algunas limitaciones que deben ser consideradas, como el número reducido de estudios elegibles y el tiempo de intervención relativamente corto observado en los estudios analizados. Luego, intervenciones más prolongadas podrían proporcionar una comprensión más detallada de los efectos del uso de dispositivos móviles en el desarrollo de competencias profesionales en estudiantes de enfermería.

## Conclusión

En conclusión, esta revisión de alcance proporcionó un mapeo de las investigaciones publicadas que relacionan el uso de dispositivos móviles en el ámbito del desarrollo de competencias profesionales de los estudiantes de enfermería. Los estudios analizados no solo revelaron las potencialidades inherentes de estos dispositivos en el entorno formativo, sino que también destacaron significativamente su contribución al perfeccionamiento de las capacidades clínicas de los estudiantes. Además, la convergencia de los resultados destaca la relevancia y el impacto positivo de los dispositivos móviles en el contexto formativo de la enfermería, ofreciendo apoyo para un enfoque más dinámico y eficaz en el proceso de aprendizaje.

Sin embargo, la revisión identificó una laguna en el conocimiento relacionada con la oportunidad aún no explorada de integrar diversas competencias profesionales por medio de una única herramienta educativa digital. Este hallazgo resalta la importancia de futuras investigaciones e iniciativas para explorar y desarrollar enfoques que maximicen el potencial de integración proporcionado por los dispositivos móviles, promoviendo una sinergia eficaz entre diferentes competencias profesionales.

 Es imperativo que estudios futuros se concentren no solo en el desarrollo de competencias aisladas, como también en la combinación equilibrada de competencias técnicas y transversales ( *hard skills* y *soft skills* ). Además, la investigación sobre los posibles impactos negativos, como la distracción y la seguridad del paciente, debe ser profundizada para garantizar que la implementación de estas tecnologías sea segura y eficaz. 

# Translation: Dispositivos móveis para o desenvolvimento das competências profissionais dos estudantes de enfermagem: revisão de escopo ^1^

**DOI:** 10.1590/1518-8345.7383.4372

**Authors:** Angélica Oliveira Veríssimo da Silva, Cristina Maria Correia Barroso Pinto, Rui Marques Vieira

**Affiliations:** 1 Universidade de Aveiro, Centro de Investigação em Didática e Tecnologia na Formação de Formadores, Departamento de Educação e Psicologia, Aveiro, Portugal.; 2 Escola Superior de Enfermagem do Porto, Centro de Investigação em Tecnologias e Serviços de Saúde e Rede de Investigação em Saúde, Porto, Portugal.

**Keywords:** Aplicativos Móveis, Smartphone, Estudantes de Enfermagem, Competência Profissional, Prática Profissional, Papel do Profissional de Enfermagem

## Abstract

**Objetivo:**

mapear a literatura científica sobre a utilização dos dispositivos móveis para o desenvolvimento das competências profissionais dos estudantes de enfermagem.

**Método:**

tratou-se de uma revisão de escopo guiada pelas recomendações do JBI. Foram incluídas seis bases de dados e a literatura cinzenta. A seleção dos estudos foi realizada através de avaliação individual e em pares. Os dados foram extraídos com base em um roteiro elaborado e apresentados de forma descritiva, tabular e gráfica.

**Resultados:**

foram identificados 264 estudos, dos quais 13 compuseram o *corpus d* e análise. Os estudos foram realizados principalmente no continente asiático. As intervenções variaram de 1 a 12 semanas, com predomínio da utilização de *Apps* . Já as competências abordadas foram principalmente as capacidades clínicas, técnicas e procedimentos inerentes à prática de enfermagem, seguidas pela tomada de decisão e resolução de problemas.

**Conclusão:**

os estudos analisados não apenas revelaram as potencialidades dos dispositivos móveis no contexto formativo, mas também destacaram a sua contribuição para o aprimoramento das capacidades clínicas, uma vez que oferecem subsídios para uma abordagem mais dinâmica e eficaz no processo de aprendizagem. A lacuna no conhecimento afigura-se na possibilidade ainda não explorada de integração de diferentes competências profissionais através de uma única ferramenta educativa digital.

## Destaques

**(1)** Dispositivos móveis têm repercussões positivas no desenvolvimento de competências.

**(2)** Lacuna do conhecimento é a ausência de uma ferramenta que integre várias competências.

**(3)** Os dispositivos móveis têm potencial para o aprimoramento das capacidades clínicas.

**(4)** Importância da integração de *hard* e *soft skills* em uma única ferramenta digital.

## Introdução

 Nos últimos tempos tem existido uma preocupação crescente com as questões ligadas à segurança e à qualidade dos cuidados, bem como com a necessidade de minimizar a ocorrência de erros evitáveis. Nesta perspectiva, é exigido que em diversas situações clínicas e diferentes contextos o enfermeiro demonstre competências para o bom julgamento clínico, a resolução de problemas e a tomada de decisão clínica ^(^ ^1^ ^-^ ^3^ ^)^ . Por esta razão, a Ordem dos Enfermeiros (OE) de Portugal, em seu Regulamento do Perfil de Competências do Enfermeiro, estabelece que para a demonstração das competências profissionais é necessária a mobilização de um conjunto de conhecimentos, habilidades e capacidades no contexto dos cuidados ^(^ ^4^ ^)^ . 

 Assim, competência é definida como “ *A competency is a holistic concept that includes knowledge, skills, attitudes and values* ” ^(^ ^5^ ^)^ . Ser competente envolve mais do que obter conhecimentos e capacidades, sobretudo implica em mobilizar os conhecimentos, as capacidades, as atitudes e os valores para responder às complexas demandas sociais e profissionais ^(^ ^5^ ^)^ . 

 No contexto de cuidados de saúde, a competência tem influência direta na saúde e na segurança dos pacientes ^(^ ^6^ ^)^ . Para a prestação de cuidados de excelência é exigido muito mais que o conhecimento, é imperativa a capacidade de pensar criticamente e aplicar na prática o conhecimento ^(^ ^7^ ^)^ . Dessa forma, o contexto de formação deverá ser um local de transformação do conhecimento teórico em prático. No entanto, a formação em enfermagem tem enfrentado diversos desafios, dentre os quais é possível citar os ambientes formativos que oferecem oportunidades precárias de transpor o conhecimento adquirido em sala de aula para a prática clínica, ora por ausência de situações clínicas, ora por falhas na supervisão clínica ^(^ ^8^ ^-^ ^9^ ^)^ . Soma-se, ainda, a crescente e exigente multiplicidade do cuidar ^(^ ^7^ ^,^ ^10^ ^)^ . Tendo em vista este complexo contexto formativo do enfermeiro, é de fundamental importância o estabelecimento de diferentes contextos e de estratégias para colmatar a lacuna entre a teoria e a prática ^(^ ^8^ ^-^ ^9^ ^)^ . 

 Os estudantes do século XXI estão em constante e crescente contato com as tecnologias digitais ^(^ ^11^ ^)^ . Neste cenário promissor e desafiador também se encontram os estudantes de enfermagem ^(^ ^8^ ^,^ ^11^ ^)^ . Logo, as tecnologias digitais são enunciadas como possibilitadoras do fornecimento de importantes informações para o processo de tomada de decisão ^(^ ^8^ ^)^ . Assim, somam-se esforços para a incorporação das tecnologias digitais, doravante designadas por recursos educativos digitais, na formação em enfermagem ^(^ ^8^ ^)^ . 

 Para a atual sociedade, globalizada, informatizada e em constante mudança, a aprendizagem móvel evidencia-se como a tecnologia digital mais promissora de aprendizagem ^(^ ^12^ ^-^ ^13^ ^)^ . Até porque os livros tornam-se obsoletos rapidamente, enquanto os dispositivos móveis fornecem informações atualizadas ^(^ ^13^ ^)^ . Devido ao seu caráter onipresente, os dispositivos móveis ^(^ ^11^ ^)^ , especialmente os *smartphones* , representam a tecnologia digital mais utilizada mundialmente ^(^ ^14^ ^)^ . Além das suas inúmeras aplicabilidades, os dispositivos móveis configuram-se como facilitadores do processo de ensino e aprendizagem, uma vez que possibilitam a flexibilidade, em outras palavras, a aprendizagem a qualquer hora e lugar ^(^ ^12^ ^,^ ^15^ ^-^ ^17^ ^)^ , por isso o conceito aprendizagem móvel. 

 A aprendizagem ativa, interativa e centrada no estudante é apontada como a que favorece o desenvolvimento das competências profissionais dos estudantes de enfermagem ^(^ ^12^ ^,^ ^18^ ^)^ . Até porque, impulsiona o estudante a pensar e a agir de forma autônoma em sua aprendizagem ^(^ ^12^ ^)^ . Tal aprendizagem é facilmente possibilitada pela utilização de conteúdos advindos dos dispositivos móveis ^(^ ^18^ ^)^ , especialmente dos aplicativos móveis ( *Apps* ). Estudos recentes evidenciaram que a utilização dos dispositivos móveis e de seus *Apps* no contexto da aprendizagem dos estudantes de enfermagem contribuíram favoravelmente para o desenvolvimento das competências profissionais ^(^ ^9^ ^,^ ^12^ ^,^ ^17^ ^-^ ^19^ ^)^ . Além disso, facilitaram o acesso à informação, aumentaram a eficiência e otimizaram o tempo ^(^ ^12^ ^)^ . Uma recente meta-análise demonstrou que a utilização dos dispositivos móveis no contexto de formação dos estudantes de enfermagem promoveu, além de conhecimento, o aprimoramento das competências profissionais, o aumento da confiança, bem como da satisfação com a aprendizagem ^(^ ^9^ ^)^ . Sobreleva-se também que a sua utilização possibilitou reduzir erros clínicos ^(^ ^12^ ^)^ . Dessa forma, a implementação de dispositivos móveis e de seus *Apps* nos currículos dos cursos de enfermagem facilitam a aprendizagem ativa e a promoção de cuidados baseados nas evidências científicas mais recentes ^(^ ^13^ ^)^ . 

Ademais, garantir que os estudantes de enfermagem recebam formação de qualidade, possibilidades de contextos formativos e estratégias que facilitem a apropriação do conhecimento e sua aplicabilidade na prática clínica constitui-se um grande desafio para os educadores e gestores. Diante do desafio apresentado, emergem questões fundamentais, as quais nortearam esta pesquisa e são: Qual o impacto da utilização de dispositivos móveis no contexto formativo da enfermagem? Quais dispositivos móveis são utilizados para o desenvolvimento das competências profissionais dos estudantes de enfermagem? Dentro desse panorama, a aprendizagem móvel emerge como uma ferramenta de grande potencial para o desenvolvimento das competências profissionais do enfermeiro. A partir da urgente necessidade do desenvolvimento das competências profissionais dos estudantes de enfermagem e das potencialidades da aprendizagem móvel, estabeleceu-se como objetivo mapear a literatura científica sobre a utilização dos dispositivos móveis para o desenvolvimento das competências profissionais dos estudantes de enfermagem.

## Método

### Tipo do estudo

 O protocolo de pesquisa da revisão de escopo foi registrado no *Open Science Framework* (OSF) ( https://doi.org/10.17605/OSF.IO/CESXK ). A síntese de conhecimento e evidências expressas por uma revisão de escopo segue a recomendação sistemática de uma revisão de literatura ^(^ ^20^ ^)^ . A sua finalidade é mapear as principais fontes e tipos de evidências sobre uma determinada temática, possibilitando identificar conceitos e teorias basilares, assim como, potenciais lacunas no conhecimento ^(^ ^21^ ^)^ . 

 O rigor metodológico e a transparência devem estar presentes em todas as etapas de uma revisão de escopo para possibilitar que o estudo seja replicado ^(^ ^22^ ^)^ . De modo a dar cumprimento ao rigor metodológico e transparência, este estudo segue as recomendações do *Joanna Briggs Institute* (JBI) ^(^ ^23^ ^)^ , sendo conduzido pelas diretrizes da *Checklist* do *Preferred Reporting Items for Systematic Reviews and Meta-analyses extension for Scoping Reviews* (PRISMA-ScR) ^(^ ^21^ ^)^ . 

Com a finalidade de identificar outros estudos ou protocolos semelhantes, realizou-se uma busca inicial, em outubro de 2023, na OSF, por ser uma plataforma aberta e gratuita para depósito de pesquisas e seus respectivos protocolos. Não foram identificados estudos relacionados com o objetivo proposto, demonstrando a pertinência desta revisão.

 Sendo assim, seguiram-se as etapas propostas pelo JBI para a concepção de uma revisão de escopo: definição da questão de investigação; estratégia de pesquisa; estabelecimento dos critérios de inclusão e exclusão; seleção de estudos; extração dos dados; síntese dos resultados ^(^ ^23^ ^)^ . 

### Cenário do estudo

 Os estudos que compõem esta revisão foram extraídos das seguintes bases de dados: Scopus, *Web of Science* (WoS), *National Library of Medicine* (PubMed), Biblioteca do Conhecimento *Online* (b-on) e SciELO. Para a integração da literatura cinzenta, efetuaram-se buscas por teses e dissertações no Repositórios Científicos de Acesso Aberto de Portugal (RCAAP). 

### Período

As buscas dos dados ocorreram entre os meses de outubro de 2023 a janeiro de 2024. Não foi delimitado período temporal como critério de busca por estudos elegíveis, uma vez que o objetivo se circunscreve em mapear o máximo de conhecimento publicado.

### Estratégia de pesquisa

Adotou-se a estratégia População, Conceito, Contexto (PCC), no qual População refere-se aos estudantes de enfermagem, Conceito remete ao desenvolvimento de competências profissionais e Contexto aos dispositivos móveis, para culminar na elaboração da questão de pesquisa: Quais são os dispositivos móveis utilizados para o desenvolvimento das competências profissionais dos estudantes de enfermagem?

### Critérios de elegibilidade

 Após a elaboração da questão de pesquisa, foram definidos os critérios de elegibilidade para inclusão e exclusão dos estudos segundo a estratégia PCC. Assim, foram incluídos estudos que: a) no que diz respeito aos participantes, englobem estudantes do ensino superior em enfermagem; b) relativamente ao conceito, estudos que se referem ao desenvolvimento das competências profissionais; c) quanto ao contexto, estudos que envolvem dispositivos móveis, aprendizagem móvel ou aplicativos. No que concerne ao tipo de estudo, foram contemplados todos os estudos primários, quantitativos, qualitativos e mistos, também estudos secundários, nomeadamente revisões de literatura e ainda a literatura cinzenta. Além disso, nesta revisão incluiu-se estudos publicados em português, inglês ou espanhol, devido ao fato de serem os idiomas com os quais os pesquisadores têm familiaridade. Acrescenta-se como critério de inclusão estudos em formato *open access.* Ressalta-se que a limitação temporal não foi um critério de exclusão. 

Foram excluídos estudos em que os participantes são enfermeiros, estudantes dos cursos técnicos ou auxiliares de enfermagem e, também, editoriais, cartas ao editor, protocolos de estudo, resumos e recomendações.

### Coleta de dados

 Postos a questão de investigação e os critérios de elegibilidade, um dos pesquisadores reuniu-se com a bibliotecária da universidade para o estabelecimento da estratégia de busca. Dessa forma, realizou-se uma busca preliminar na base de dados Scopus com os descritores *“nursing students”, “mobile devices”* e *“professional competence”* para identificar, nos títulos e resumos, os descritores mais utilizados sobre a temática. 

 Na sequência, os descritores encontrados foram combinados de diferentes formas através de operadores booleanos *AND* e *OR* , resultando nas frases booleanas, conforme a Figura 1 , configurando-se como a estratégia de busca para as diferentes bases de dados. 

**Figura 1:** - Estratégia de busca. Aveiro, Portugal, 2023

Base de dados	Estratégia de busca	Resultados
Scopus	( TITLE-ABS-KEY ( “nurs* education” OR “nurs* students” ) AND TITLE-ABS-KEY ( “mobile device*” OR “mobile technolog*” OR “mobile application*” OR “smartphone*” OR “mobile learning” ) AND TITLE-ABS-KEY ( “skill acquisition” OR “nurs* skills” OR “professional competence” OR “professionalism” OR “professional practice” OR “clinical competence” ) )	85
WoS	((TS=(“nurs* education” OR “nurs* students”)) AND TS=(“mobile device*” OR “mobile technolog*” OR “mobile application*” OR “smartphone*” OR “mobile learning” )) AND TS=(“skill acquisition” OR “nurs* skills” OR “professional competence” OR “professionalism” OR “professional practice” OR “clinical competence”)	27
PubMed	*((“nursing education”[Title/Abstract] OR “nursing students”[Title/Abstract]) AND (“mobile devices”[Title/Abstract] OR “mobile technology”[Title/Abstract] OR “mobile application”[Title/Abstract] OR “smartphone”[Title/Abstract] OR “mobile learning”[Title/Abstract])) AND (“skill acquisition”[Title/Abstract] OR “nursing skills”[Title/Abstract] OR “professional competence”[Title/Abstract] OR “professionalism”[Title/Abstract] OR “professional practice”[Title/Abstract] OR “clinical competence”[Title/Abstract])*	20
b-on	AB ( “nurs* education” OR “nurs* students” ) AND AB ( “mobile device*” OR “mobile technolog*” OR “mobile application*” OR “smartphone*” OR “mobile learning” ) AND AB ( “skill acquisition” OR “nurs* skills” OR “professional competence” OR “professionalism” OR “professional practice” OR “clinical competence” )	33
SciELO	#2 (ab:(estudante* de enfermagem *OR* educação em enfermagem )) *AND* (ab:(dispositivo* move* *OR* aprendiza* move* *OR* smartphone* *OR* app*))	14
RCAAP	*Title “nursing students” AND “mobile devices” OR “mobile technology” OR “mobile application” OR “smartphone” OR “mobile learning” AND Abstract “nursing students” AND “mobile devices” OR “mobile technology” OR “mobile application” OR “smartphone” OR “mobile learning” AND Subject “nursing students” AND “mobile devices” OR “mobile technology” OR “mobile application” OR “smartphone” OR “mobile learning”*	85

### Tratamento e análise de dados

 Os resultados da pesquisa efetuada foram exportados para o *software* *Mendeley Reference Manager version* 2.80.1, sendo que todos os estudos duplicados foram eliminados. Na sequência, dois revisores independentes fizeram a leitura dos títulos e dos resumos para verificação do cumprimento dos critérios de inclusão e confirmar a elegibilidade dos estudos ^(^ ^24^ ^)^ . As eventuais divergências entre os dois revisores foram resolvidas por consenso, ou recorrendo a um terceiro revisor. Os estudos que respeitaram os critérios de inclusão definidos, foram submetidos a leitura do texto integral. Posteriormente, os estudos foram importados para o *Qualitative Data Analysis Software* ( *webQDA* ) para a sua respectiva análise qualitativa. Os dados estão apresentados de acordo com o objetivo desta revisão, em formato gráfico, tabular e texto descritivo. 

 Após o estabelecimento do *corpus* , dois revisores independentes realizaram a extração dos dados através de um instrumento, segundo as recomendações da JBI. O instrumento foi desenvolvido pelo grupo de pesquisadores para extração das seguintes variáveis: autor, título do estudo, país de realização do estudo, ano de publicação, tipo de estudo, objetivo, estratégia/ferramenta utilizada, tempo de intervenção, competência a ser desenvolvida e resultados alcançados. 

### Aspectos éticos

A pesquisa utilizou apenas dados de domínio público, por isso não foi necessário solicitar um parecer a um Comitê de Ética em Pesquisa. Ressalta-se que os direitos autorais foram respeitados por meio da correta citação e referência bibliográfica.

## Resultados

 A pesquisa nas cinco bases de dados e um repositório possibilitou identificar 264 estudos potencialmente relevantes. A aplicação dos critérios de exclusão, nomeadamente língua, tipo de estudo e ausência de texto integral formato *Open Access* permitiu excluir 112 estudos. Dos 152 estudos remanescentes, 23 foram excluídos devido a duplicidade. Os demais 129 foram então submetidos à leitura dos títulos e resumos, sendo assim, excluídos 93. Na fase seguinte, os 36 estudos restantes foram submetidos à leitura integral, e destes, foram excluídos 23. À vista disso, através da estratégia de busca estabelecida, a pesquisa nas bases de dados e repositório possibilitou identificar 264 estudos. A aplicação de critérios de inclusão e de exclusão, acrescida da análise minuciosa dos pesquisadores, proporcionou identificar uma amostra documental de 13 estudos, configurando o *corpus* de análise desta revisão. 

 Os estudos elegíveis encontram-se representados na Figura 2 . 

**Figura 2:** - Fluxograma PRISMA ^(^ ^24^ ^)^ . Aveiro, Portugal, 2023

 A lista dos países constituintes deste *corpus* é liderada pelo continente asiático com nove estudos, sendo cinco da Coreia, três da China e um do Irã. Em contrapartida, o continente europeu é representado por três estudos, sendo dois pertencentes à Noruega e um à Finlândia. Por fim, o continente americano é representado apenas com um estudo, pelo Brasil. 

 No que diz respeito à distribuição dos estudos por ano de publicação, verificou-se que em 2018 foram publicados quatro artigos, representando o ano com maior número de publicações (30,76%). Quanto aos aspectos metodológicos das pesquisas incluídas destacam-se quatro ensaios clínicos randomizados controlados e quatro do tipo quase-experimental. A caracterização dos estudos quanto ao título, país, ano de publicação, tipo de estudo e objetivo encontra-se sintetizada na Figura 3 . 

 Com vistas à sistematização dos resultados, os dados foram organizados em forma de quadro ( Figura 4 ) para ressaltar a estratégia utilizada em cada estudo. Além disso, apresenta-se a finalidade em utilizá-la, ou seja, a competência profissional que a estratégia buscou desenvolver, o tempo de intervenção e os seus respectivos resultados. 

**Figura 3:** - Caracterização dos estudos quanto ao título, país, ano de publicação e tipo de estudo. Aveiro, Portugal, 2023

**ID**	**Título**	**País/Ano**	**Tipo de Estudo**	**Objetivo**
A1	*A smartphone application to educate undergraduate nursing students about providing care for infant airway obstruction*	Coreia, 2017	Quase-experimental	Desenvolver um *App* para *smartphone* e avaliar a sua eficácia quanto ao conhecimento, às capacidades e à confiança dos estudantes de enfermagem.
A2	*Configuration of Mobile Learning Tools to Support Basic Physical Assessment in Nursing Education- Longitudinal Participatory Design Approach*	Noruega, 2021	Qualitativo e longitudinal	Projetar um conjunto de ferramentas *mobile learning* para apoiar a aprendizagem dos estudantes de enfermagem.
A3	*Effectiveness of mobile cooperation intervention on students’ clinical learning outcomes: A randomized controlled trial*	Finlândia, 2018	Ensaio clínico randomizado e controlado	Avaliar a eficácia da intervenção de cooperação móvel para melhorar a competência e a autoeficácia dos estudantes de enfermagem.
A4	*Effects of a skill demonstration video delivered by smartphone on facilitating nursing students’ skill competencies and self-confidence: A randomized controlled trial*	China, 2018	Ensaio clínico randomizado e controlado	Examinar os efeitos de um vídeo em *smartphone* na competência e na confiança dos estudantes de enfermagem.
A5	*Effects of Nursing Students’ Practices using Smartphone Videos on Fundamental Nursing Skills, Self-efficacy, and Learning Satisfaction in South Korea*	Coreia, 2017	Quase-experimental	Investigar os efeitos da aprendizagem por meio do uso de gravação de vídeo em *smartphones* na autoavaliação dos estudantes.
A6	*Effects of using mobile device-based academic electronic medical records for clinical practicum by undergraduate nursing students- A quasi-experimental study.*	Coreia, 2018	Quase-experimental	Examinar o efeito de um *App* no estágio clínico dos estudantes de enfermagem.
A7	*Evaluation of mobile learning for the clinical practicum in nursing education- application of the FRAME model*	China, 2019	Experimental	Avaliar a percepção dos estudantes de enfermagem sobre o uso de dispositivos móveis na prática clínica.
A8	*Factors associated with changes in students’ self-reported nursing competence after clinical rotations: A quantitative cohort study*	Noruega, 2023	Quantitativo de coorte	Explorar as mudanças na competência de enfermagem, os fatores associados às mudanças após as rotações clínicas e se um conjunto de ferramentas de aprendizagem móvel apoia as mudanças no uso confiante das capacidades básicas de avaliação física.
A9	*Use of digital applications in the medicament calculation education for nursing*	Brasil, 2016	Experimental	Avaliar a influência do uso de aplicativos digitais no ensino de cálculo de medicamentos para estudantes de enfermagem.
A10	*Mobile-Based Video Learning Outcomes in Clinical Nursing Skill Education: A Randomized Controlled Trial*	Coreia, 2016	Ensaio clínico randomizado e controlado	Identificar os efeitos dos videoclipes em dispositivos móveis sobre a motivação para o aprendizado, a competência e a satisfação dos estudantes de enfermagem, e explorar as relações entre essas variáveis.
A11	*Using Video Feedback Through Smartphone Instant Messaging in Fundamental Nursing Skills Teaching: Observational Study*	China, 2019	Observacional	Explorar os possíveis efeitos do *feedback* em vídeo por meio de mensagens em *smartphones* no ensino de capacidades fundamentais de enfermagem para estudantes de enfermagem.
A12	*The Effect of Team-based Training Through Smartphone Applications on Nursing Students’ Clinical Skills and Problem-Solving Ability*	Irã, 2022	Quase-experimental	Determinar o efeito de um programa educacional, baseado em equipe, por meio de aplicativos de *smartphone,* na capacidade de resolução de problemas e nas capacidades clínicas dos estudantes de enfermagem.
A13	*The Effects of an Interactive Nursing Skills Mobile Application on Nursing Students’ Knowledge, Self-efficacy, and Skills Performance: A Randomized Controlled Trial*	Coreia, 2018	Ensaio clínico randomizado e controlado	Avaliar o efeito de um *App* de capacidades de enfermagem para estudantes de enfermagem.

**Figura 4:** - Caracterização dos estudos quanto à estratégia utilizada, à competência desenvolvida, ao tempo de intervenção e os respectivos resultados. Aveiro, Portugal, 2023

**ID**	**Estratégia**	**Competência**	**Tempo de intervenção**	**Resultados**
A1	*App* para desobstrução de vias aéreas de crianças *(smartphone)*	Capacidade clínica (desobstrução de vias aéreas)	6 semanas	Diferença estatística significativa do grupo experimental nas capacidades clínicas e na confiança no desempenho. Relataram maior satisfação com a aprendizagem.
A2	Ferramentas *Mobile-Learning* baseadas em: Simulação clínica virtual, MOOC, vídeos e *podcast*	Capacidade clínica (exame físico)	12 semanas	Os estudantes consideraram o conjunto de ferramentas benéfico para apoiar o desenvolvimento das competências.
A3	*App* *Study@Campus* : diário de aprendizagem, cooperação entre estudantes e professores e *feedback (smartphone)*	Capacidades clínicas ( *Nurse Competence Scale* ) e autoeficácia	5 semanas	Não revelou diferenças estatísticas entre os dois grupos. Efeito significativo na satisfação dos estudantes.
A4	Vídeo de demonstração de capacidades *(smartphone)*	Conhecimento, capacidade clínica e confiança (cateterismo urinário)	2 semanas	Diferenças com significância estatística tanto no conhecimento, quanto nas capacidades clínicas e na satisfação com a aprendizagem do grupo experimental.
A5	Vídeo de prática de administração de medicação por via intramuscular ( *smartphone* )	Capacidade clínica, autoeficácia e satisfação (administração de medicamentos)	2 semanas	Efeitos significativos e positivos na melhoria da competência e na satisfação com a aprendizagem do grupo experimental.
A6	*App Academic electronic medical record* (AEMR) ( *smartphone* )	Tomada de decisão	4 semanas	O grupo experimental apresentou aumento significativo no domínio de conhecimentos e de competências. Não houve diferença com significância estatística no pensamento crítico entre o grupo experimental e controle. O grupo experimental apresentou maior satisfação com a aprendizagem.
A7	*App* 1- avaliação de capacidades clínicas *App* 2- vídeos de procedimentos clínicos ( *tablet* )	Capacidades clínicas	^*^	Satisfação dos participantes com a prática de aprendizagem móvel.
A8	Conjunto de ferramentas no programa Canvas *(smartphone e tablet)*	Capacidade clínica e autoeficácia (exame físico)	8 semanas	Diferenças estatísticas significativas na competência e na confiança após o uso do conjunto de ferramentas.
A9	*App* CalcMed *(smartphone)*	Cálculo de medicamentos	^*^	Influenciou positivamente a aprendizagem e a segurança na realização do cálculo de medicamentos.
A10	Vídeo cateterismo urinário *(smartphone)*	Capacidade clínica (cateterismo urinário)	3 semanas	Grupo experimental apresentou níveis significativamente mais elevados de motivação e satisfação com a aprendizagem.
A11	Vídeos de demonstração de capacidades clínicas para *feedback (smartphone)*	Capacidades clínicas (arrumação da cama, procedimentos assépticos, sinais vitais, oxigenoterapia),	8 semanas	Grupo experimental apresentou pontuações maiores no exame final das capacidades clínicas e, também, maior satisfação com a estratégia utilizada.
A12	*App Telegram* : materiais didáticos. *Team Based Learn* *(smartphone)*	Resolução de problemas	12 semanas	As pontuações médias aumentaram mais no grupo experimental.
A13	*App* ICNS: simulação clínica *(smartphone)*	Conhecimento, capacidade clínica e autoeficácia (sinais vitais, medicação intravenosa, sondagem gástrica e aspiração endotraqueal)	1 semana	O grupo experimental apresentou um valor significativamente maior de conhecimento, autoeficácia e capacidades clínicas. Evidenciaram maior satisfação com a aprendizagem.

* Não é possível identificar o tempo exato de intervenção

## Discussão

A acessibilidade e a versatilidade dos dispositivos móveis oferecem oportunidades significativas para a aprendizagem. Em vista disto, esta revisão teve como objetivo mapear a literatura científica sobre a utilização dos dispositivos móveis para o desenvolvimento das competências profissionais dos estudantes de enfermagem, identificando as diferentes estratégias utilizadas para esse fim.

 Vale salientar que os estudos concentraram-se no continente asiático, especialmente na Coreia ^(^ ^18^ ^,^ ^25^ ^-^ ^28^ ^)^ e na China ^(^ ^29^ ^-^ ^31^ ^)^ , que emergiram como líderes globais em investimentos e avanços tecnológicos. A China, por meio de um programa nacional, tem empreendido grandes esforços para o desenvolvimento científico e tecnológico, com o objetivo de fomentar uma força de trabalho altamente qualificada ^(^ ^32^ ^)^ . Além disso, a análise dos estudos selecionados revela um aumentado significativo no interesse pelo tema, com um crescimento de 69% no número de artigos publicados a partir de 2018. 

 Ademais, uma reflexão importante refere-se ao tempo de intervenção, que variou entre 1 ^(^ ^18^ ^)^ e 12 semanas ^(^ ^33^ ^-^ ^34^ ^)^ . Curiosamente, o tempo de intervenção aparentemente não determinou a eficácia da estratégia utilizada, uma vez que o estudo realizado em apenas 1 semana ^(^ ^18^ ^)^ demonstrou resultados mais satisfatórios do grupo experimental em relação ao controle. Em contrapartida, o estudo cuja intervenção foi realizada em 5 semanas ^(^ ^35^ ^)^ não apresentou diferenças estatísticas significativas entre os grupos experimental e controle. Isto sugere que a relação entre o tempo de intervenção e a aquisição de competências deve ser cuidadosamente considerada, uma vez que competências podem ser desenvolvidas por meio de contextos formativos apropriados ^(^ ^3^ ^)^ . 

 Nesse ínterim, os estudantes demonstraram níveis elevados de satisfação em dez estudos, ao utilizarem estratégias por meio dos seus dispositivos móveis ^(^ ^18^ ^,^ ^25^ ^-^ ^29^ ^,^ ^31^ ^,^ ^33^ ^,^ ^35^ ^)^ . Um estudo no Reino Unido revelou benefícios como aquisição de conhecimento e de confiança, além da redução da ansiedade na prática clínica ^(^ ^19^ ^)^ . Uma meta-análise corroborou esses achados, indicando que a aprendizagem móvel melhora as competências, os conhecimentos, a satisfação com a aprendizagem e a confiança dos estudantes ^(^ ^9^ ^)^ . 

 O tipo de intervenção realizada é outro ponto relevante: nota-se que dos 13 estudos, sete utilizaram *Apps* ^(^ ^18^ ^,^ ^25^ ^,^ ^27^ ^,^ ^30^ ^,^ ^34^ ^-^ ^36^ ^)^ , quatro utilizaram vídeos para demonstração de capacidades clínicas ^(^ ^26^ ^,^ ^28^ ^-^ ^29^ ^,^ ^31^ ^)^ e dois utilizaram diferentes ferramentas em dispositivos móveis, tais como *Massive Open Online Course* (MOOC), vídeos, *podcasts* e ferramentas no programa Canvas ^(^ ^33^ ^,^ ^37^ ^)^ . 

 A maioria dos *Apps* foi especificamente voltada para o desenvolvimento de competências profissionais ^(^ ^18^ ^,^ ^25^ ^,^ ^27^ ^,^ ^30^ ^,^ ^36^ ^)^ . Enquanto um estudo na Finlândia utilizou o *App Study@Campus* , que incluía um recurso de *chat* para interação e *feedback* imediato ^(^ ^35^ ^)^ , no Irã, o *Telegram* foi utilizado para disponibilização de conteúdos didáticos e partilha de dúvidas ^(^ ^34^ ^)^ , mostrando-se eficaz no aprimoramento de capacidades clínicas e resolução de problemas. Este estudo fortalece a ideia que a comunicação através de mensagens instantâneas, pelas redes sociais, configura-se como um espaço de partilha ao promover a discussão em “tempo real” ^(^ ^38^ ^)^ . 

 Além de que, os vídeos destacaram-se como uma estratégia importante para o desenvolvimento de competências profissionais, devido à possibilidade de revisão contínua do conteúdo. Dos 13 estudos, dez focaram o desenvolvimento de competências clínicas, nomeadamente técnicas e procedimentos de que os enfermeiros devem ter conhecimento, habilidades e capacidades para os realizar ^(^ ^18^ ^,^ ^25^ ^-^ ^26^ ^,^ ^28^ ^-^ ^31^ ^,^ ^33^ ^,^ ^35^ ^,^ ^37^ ^)^ , enquanto três abordaram competências como tomada de decisão ^(^ ^27^ ^)^ , resolução de problemas ^(^ ^34^ ^)^ e cálculo de medicamentos ^(^ ^36^ ^)^ . Conquanto que as intervenções não exploraram profundamente as potencialidades dos dispositivos móveis para o desenvolvimento de múltiplas competências, focando-se em desenvolver capacidades clínicas ou outras competências específicas. 

 Considerando a abrangência do conceito de competência, houve a necessidade de segmentação entre competências técnicas ( *hard skills* ) e transversais ( *soft skills* ) ^(^ ^39^ ^)^ . As *soft skills* são fundamentais para promover o desempenho eficaz em diversos contextos profissionais e sociais ^(^ ^3^ ^)^ , além de englobarem as capacidades cognitivas, metacognitivas, interpessoais, intelectuais e práticas, bem como valores éticos ^(^ ^3^ ^,^ ^40^ ^)^ . Enquanto as *soft skills* são consideradas «transferíveis», pois podem ser aplicadas em diversas situações e contextos, as *hard skills* , relacionadas aos conhecimentos técnicos e científicos específicos, formam a base técnica necessária para desempenhar funções especializadas ^(^ ^3^ ^,^ ^39^ ^)^ . A combinação equilibrada de *soft skills* e *hard skills* é essencial para a excelência profissional. 

 Sendo assim, capacidades clínicas ( *hard skills* ) eficazes são essenciais para a promoção de cuidados seguros e de qualidade ^(^ ^41^ ^-^ ^42^ ^)^ . Os estudantes de enfermagem precisam ser estimulados ao desenvolvimento das *soft skills* . Para isso, deverão ser incentivados a pensar de forma crítica ^(^ ^1^ ^,^ ^43^ ^-^ ^44^ ^)^ e desenvolver as competências de julgamento clínico, resolução de problemas e tomada de decisão ^(^ ^1^ ^,^ ^45^ ^-^ ^48^ ^)^ , contextos educativos estes que estimulam o pensamento crítico e contribuem para a formação de profissionais mais capacitados e adaptáveis ^(^ ^1^ ^,^ ^44^ ^)^ . Além disso, o desenvolvimento do julgamento clínico torna-se fundamental para que os estudantes de enfermagem possam avaliar eficientemente os cenários clínicos para a tomada de decisão. Neste sentido, é fundamental que os contextos formativos sejam estruturados de maneira a oferecer uma abordagem holística no desenvolvimento das *hard* e *soft skills* , preparando os estudantes não apenas para as demandas técnicas, mas também para os desafios cognitivos e decisórios inerentes à prática profissional em enfermagem ^(^ ^46^ ^,^ ^49^ ^)^ . Dessa forma, os esforços deverão ser orientados para o desenvolvimento/fortalecimento do pensamento crítico, do julgamento clínico, da resolução de problemas e da tomada de decisão ^(^ ^1^ ^-^ ^3^ ^,^ ^44^ ^,^ ^50^ ^)^ . 

 Nesse contexto, os dispositivos móveis surgem como uma abordagem econômica, acessível e eficaz para aprimorar as competências profissionais ^(^ ^41^ ^)^ . Eles permitem um ambiente de aprendizagem flexível, com acesso aos conteúdos, a qualquer hora e em qualquer lugar ^(^ ^25^ ^,^ ^41^ ^-^ ^42^ ^,^ ^51^ ^)^ . A possibilidade de revisitar o conhecimento continuamente, combinada com a familiaridade e o gosto pela tecnologia, torna os dispositivos móveis eficazes no desenvolvimento de competências profissionais ^(^ ^52^ ^-^ ^53^ ^)^ e confiança dos estudantes ^(^ ^19^ ^,^ ^41^ ^)^ . A integração de estratégias de ensino através dos dispositivos móveis pode efetivamente engajar e aprimorar competências como o pensamento crítico, julgamento clínico e tomada de decisão ^(^ ^53^ ^-^ ^55^ ^)^ . À vista disso, os dispositivos móveis não apenas dinamizam, mas também complementam o processo de aprendizagem. Ademais, os recursos educativos digitais disponibilizados pelos dispositivos móveis, tais como *MOOC* ’s, *Apps* , vídeos, animações e jogos podem ser utilizados tanto em interfaces presenciais quanto remotas, podendo contribuir para uma aprendizagem mais imersiva e prática ^(^ ^56^ ^)^ . 

 Apesar das potencialidades dos dispositivos móveis, é necessário ressaltar possíveis aspectos negativos na utilização dos dispositivos móveis na formação dos estudantes de enfermagem ^(^ ^42^ ^,^ ^56^ ^-^ ^57^ ^)^ , como a distração que pode comprometer a eficiência e a segurança no atendimento. Uma revisão de literatura realizada na Austrália destacou esses impactos negativos, incluindo violações da segurança do paciente e no controle de infecção ^(^ ^57^ ^)^ . 

Em síntese, as principais contribuições desta revisão são os desfechos positivos evidenciados em todos os estudos analisados, confirmando que os dispositivos móveis influenciam significativamente o desenvolvimento das competências profissionais dos estudantes de enfermagem. No entanto, é necessário conduzir pesquisas mais abrangentes para explorar a sinergia entre as competências profissionais e as transversais, reconhecendo que a formação em enfermagem abrange não apenas as capacidades técnicas, mas também o desenvolvimento holístico do estudante.

Por fim, este estudo apresenta algumas limitações que devem ser consideradas, como o número reduzido de estudos elegíveis e o tempo de intervenção relativamente curto observado nos estudos analisados. Logo, intervenções mais prolongadas poderiam proporcionar uma compreensão mais detalhada dos efeitos da utilização dos dispositivos móveis no desenvolvimento das competências profissionais nos estudantes de enfermagem.

## Conclusão

Em conclusão, esta revisão de literatura proporcionou o mapeamento das pesquisas publicadas que relacionam a utilização de dispositivos móveis no âmbito do desenvolvimento das competências profissionais dos estudantes de enfermagem. Os estudos analisados não apenas revelaram as potencialidades inerentes desses dispositivos no ambiente formativo, mas também destacaram de maneira significativa sua contribuição para o aprimoramento das capacidades clínicas dos estudantes. Além disso, a convergência dos resultados destaca a relevância e o impacto positivo dos dispositivos móveis no contexto formativo da enfermagem, oferecendo subsídios para uma abordagem mais dinâmica e eficaz no processo de aprendizagem.

No entanto, a revisão identificou uma lacuna no conhecimento relacionada à oportunidade ainda não explorada de integrar diversas competências profissionais através de uma única ferramenta educativa digital. Este achado ressalta a importância de futuras pesquisas e iniciativas no sentido de explorar e desenvolver abordagens que maximizem o potencial de integração proporcionado pelos dispositivos móveis, promovendo uma sinergia eficaz entre diferentes competências profissionais.

 É imperativo que estudos futuros se concentrem não apenas no desenvolvimento de competências isoladas, como também na combinação equilibrada de competências técnicas e transversais ( *hard skills* e *soft skills* ). Além disso, a investigação sobre os impactos negativos potenciais, como a distração e a segurança do paciente, deve ser aprofundada para garantir que a implementação dessas tecnologias seja segura e eficaz.
